# Synthesis, photochemical and biological evaluation of novel photoswitchable glycomimetic ligands of *Pseudomonas aeruginosa* LecB

**DOI:** 10.1039/d5ra06897e

**Published:** 2025-12-12

**Authors:** Shapla Bhattacharya, Giorgia Tempra, Alessio Colleoni, Carlo Matera, Rossella Castagna, Emilio Parisini

**Affiliations:** a Department of Biotechnology, Latvian Institute of Organic Synthesis Aizkraukles 21 LV-1006 Riga Latvia emilio.parisini@osi.lv; b Faculty of Materials Science and Applied Chemistry, Riga Technical University Paula Valdena 3 LV-1048 Riga Latvia; c Department of Pharmaceutical Sciences, University of Milan Via Luigi Mangiagalli 25 20133 Milan Italy; d Department of Chemistry, Biology and Biotechnology, University of Perugia Via Elce di Sotto 8 06123 Perugia Italy; e Department of Chemistry, Materials and Chemical Engineering “G. Natta”, Politecnico di Milano Piazza Leonardo da Vinci 32 20133 Milano Italy rossella.castagna@polimi.it; f Department of Chemistry “G. Ciamician”, University of Bologna Via P. Gobetti 85 40129 Bologna Italy

## Abstract

Bacterial multidrug resistance (MDR) poses a major threat to global health. The continued use of antibiotics, combined with genetic variations and exposure to nosocomial infections, has led to the selection and spread of multidrug-resistant bacteria. In recent years, photopharmacology has emerged as a strategy to combat MDR by enabling precise, light-controlled spatiotemporal modulation of the biological activity of photo-switchable compounds. Among different microbial species, *Pseudomonas aeruginosa* is a prominent bacterium involved in acute and chronic lung infections, posing a significant health concern, particularly among hospitalized and immunocompromised patients. The bacterium's capacity to form biofilms, a key factor in the development of MDR, is closely linked to the activity of the virulence factor LecB, a carbohydrate-binding protein with a well-documented role in biofilm formation. In this study, we report the design, synthesis and biological evaluation of two novel photoswitchable LecB modulators, photofucose-1 and photofucose-2. Isothermal Titration Calorimetry (ITC) analysis revealed that photofucose-2 binds LecB with high affinity, exhibiting a distinct difference in dissociation constants (*K*_d_) between its *cis* and *trans* isomers. Moreover, we determined the X-ray crystal structure of the LecB-photofucose-2 complex, offering insights into its binding mechanism. These findings lay the groundwork for the rational, structure-based design of novel light-responsive compounds targeting LecB and represent a potential new avenue in the development of innovative strategies to combat bacterial resistance.

## Abstract 2

Photoswitchable targeting of LecB with structural elucidation of the photofucose-2 complex.

## Graphical abstract

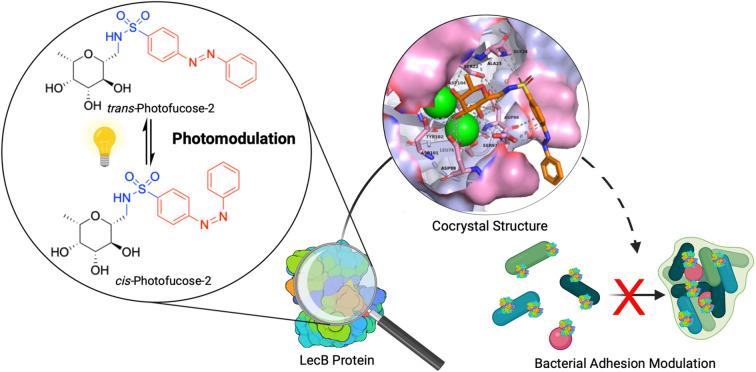

## 1 Introduction

The development of multi-drug resistance (MDR) among various bacterial species is a global health crisis, contributing to hundreds of thousands of deaths annually.^1^ Several projections estimate that the number of deaths caused by bacterial infections will reach 10 million by 2050. A recent study by the World Health Organization (WHO) revealed that antibiotic-resistant bacteria cause over half a million infections annually in the European Union, resulting in more than 30 000 deaths directly attributable to these infections.^2^*Pseudomonas aeruginosa* (*PA*) is a ubiquitous, opportunistic Gram-negative bacterium commonly found in the environment worldwide. It is a frequently encountered pathogen, particularly associated with both acute and chronic lung infections.^3^*PA* poses a significant threat, particularly to hospitalized and immunocompromised patients, often worsening their clinical conditions or causing severe diseases.^4^ This bacterium can infect tissues, including wounds, the urinary tract and the respiratory tract. In recent years, numerous studies have underscored the clinical significance of *PA* infections, leading to its classification as a high-priority pathogen by the WHO in 2024.^5^ The development of drug resistance in *PA* arises from multiple drug-neutralizing mechanisms, including the production of beta-lactamases, alterations in porin channels and efflux pumps, and the bacterium's ability to form biofilms. In biofilms, bacteria are embedded in a self-produced matrix that protects them against host immune defences and antibiotic treatment.^6,7^ The process of biofilm formation involves several factors, including bacterial cell adhesion proteins known as adhesins, such as lectins. These proteins recognize specific sugar moieties present on the surface of neighbouring bacterial cells and host cells.^8^*PA* possesses two crucial lectins, LecA and LecB (a.k.a. PA-IL and PA-IIL), which are both tetravalent carbohydrate-binding proteins. The expression of these two virulence factors is regulated by quorum sensing^9^ and plays a pivotal role in biofilm formation. LecA and LecB proteins have been observed to cross-link glycoconjugates on host cells or tissues with bacterial lipopolysaccharide and exopolysaccharides, thereby stabilizing the matrix and safeguarding the integrity of the biofilm.^10,11^ Since lectin-mediated recognition and adhesion between *PA* and host cells are critical early steps in the pathogenesis of infection, inhibiting this interaction with exogenous compounds offers a potential strategy to prevent biofilm formation or disrupt established biofilms.^3^ The exact mechanism by which lectins contribute to biofilm formation is not fully understood yet. It has been hypothesized that these tetravalent lectins act as bridging agents, facilitating glycan-mediated bacterial adhesion to the cellular glycocalyx of the host tissue.^12^ LecB protein consists of four subunits, each exhibiting a high degree of specificity for l-fucose and its derivatives^13^ as well as for d-mannose, albeit with a lower binding affinity.^14^ Since LecB is localized in the extracellular environment on the outer membrane, the Gram-negative bacterial cell envelope, which typically poses a significant challenge for many antibiotics with intracellular targets, does not impede the targeting of LecB.^15^ The pioneering work of Johansson *et al.* demonstrated that glycopeptide dendrimers targeting LecB resulted in the complete inhibition and dispersion of biofilms, which clearly suggests that this lectin is a valuable target for the development of *PA* biofilm inhibitors.^16^ Furthermore, LecB is involved in the assembly of pili on the bacterial cell surface. Inhalation of an aerosol containing fucose and galactose, the ligands of LecB and LecA, respectively, has been shown to reduce bacterial loads in infected human airways and in mice.^17^ LecB mediates contact between bacterial cells through cell-surface glycoconjugates.^18^ This biofilm-reinforcing property of lectins, resulting from their multivalency, can be blocked by inhibiting the carbohydrate binding sites.^12^

Light-triggered approaches are an emerging field of interest due to their potential as a non-invasive, precise method with spatial and temporal control and with multifunctional properties.^19,20^ They offer a promising alternative to traditional therapies and have been applied to a variety of cellular targets and protein–ligand and protein–protein interactions with both *in vitro* and *in vivo* applications.^21–24^ Such therapies provide high spatiotemporal resolution, are generally non-invasive at various wavelengths, are inert to most elements of living systems, and do not contaminate samples. Additionally, the wavelength and intensity of light can be precisely controlled, offering further advantages over traditional approaches. The ability to deliver a light stimulus with high spatiotemporal precision has led to the rapid expansion of light-based strategies to combat bacterial proliferation and biofilm formation.^25,26^ Compared to traditional pharmacotherapy, the distinct advantage of photoswitchable ligands is that light can confine the antibacterial action to when and where it is needed.^2^ Photoswitchable ligands can undergo reversible changes in their properties, such as affinity, structure, or reactivity, upon exposure to light. These ligands feature photoactive functional groups that undergo photoisomerization, a reversible change between isomeric forms triggered by light absorption. Incorporation of an azobenzene moiety into an antibacterial drug allows for light-controlled regulation of its activity, representing an innovative approach for targeting novel pathways in photopharmacology.^27^ Photoswitchable antimicrobials offer several advantages over traditional drugs in combatting biofilm formation and bacterial colonization.^28,29^ One key advantage is the ability to achieve spatiotemporal control, allowing for localized treatment and reducing systemic exposure, which helps minimize side effects on healthy tissues. Moreover, reversible photoswitchable antimicrobials are less prone to induce drug resistance mechanisms because they can be switched between inactive and active forms using light.^30–32^ This adds a layer of control over their activity and reduces the constant selective pressure that traditional antimicrobials often exert, which commonly leads to resistance. Indeed, since the drug is only activated when necessary and can be released in the environment in an inactive form, bacteria have less time and opportunities to develop resistance mechanisms.

Building on the promising potential of light-triggered approaches for targeting bacterial proteins, researchers have developed a range of photoswitchable molecules and peptides to modulate protein–protein and protein–ligand interactions.^32,33^ These strategies exemplify the power of photoswitchable systems in studying and manipulating protein interfaces dynamically. Photocleavable glycomimetics, such as surface-bound mannosides, have demonstrated light-dependent regulation of bacterial adhesion and spatial organization providing valuable tools to study biofilm structure–function relationships and quorum sensing.^33^

A ligand-based approach has been followed in the design of azobenzene-integrated glycomaterials, to enable precise photocontrol over lectin binding. The synthesis of bis-glycosylated azobenzenes has been also devised.^34^ A divalent ligand based on the arylazopyrazole photoswitch has recently been proposed for the model lectin wheat germ agglutinin (WGA). In its *trans* conformation, the ligand is capable of bridging adjacent binding sites on the lectin, resulting in a chelating binding mode.^35^ Bacterial lectins such as *Escherichia coli* fimbrial lectin (FimH) and *Pseudomonas aeruginosa* LecA have been targeted with azobenzene glycomimetic inhibitors based on mannose and galactose, respectively.^36,37^ An elegant supramolecular approach to targeting LecA and LecB has also been proposed, involving a library of photoswitchable Janus glycodendrimer micelles that function as multivalent inhibitors of these lectins.^38^ However, although the micelle design incorporated azobenzenes, no significant modulation of their binding potency was observed upon UV irradiation, likely due to poor photoisomerization efficiency of the glycodendrimer. To overcome this issue further effort needs to be dedicated to the ligand design to enhance both ligand binding affinity and light-response.

In this study we designed, synthesized and characterized two novel low molecular weight photoswitchable inhibitors of the *PA* LecB protein, derived from fucose: photofucose-1 and photofucose-2. To our knowledge, these represent the first structurally characterized small-molecule ligands capable of light-controlled modulation of LecB binding affinity. Previous work by Hu *et al.*^38^ described micelle-forming systems incorporating photoswitchable fucosides targeting LecB and LecA; however, no light-dependent change in lectin binding was observed, likely due to limited photoisomerization efficiency. We expressed and purified *PA* LecB and subsequently tested the binding affinity of both molecules for the target protein by isothermal titration calorimetry (ITC). Photofucose-2 exhibited micromolar potency towards LecB, with a significant difference in activity between its *cis* and the *trans* isomers. Additionally, we solved the crystal structure of LecB in complex with photofucose-2 at 1.4 Å resolution, representing a key step towards the rational design of more efficient ligands. This study demonstrates that light can modulate ligand–lectin binding affinity, laying the foundation for the development of other photoswitchable drugs based on this scaffold.

## 2 Results and discussion

### 2.1 Design strategy

**Scheme 1:**
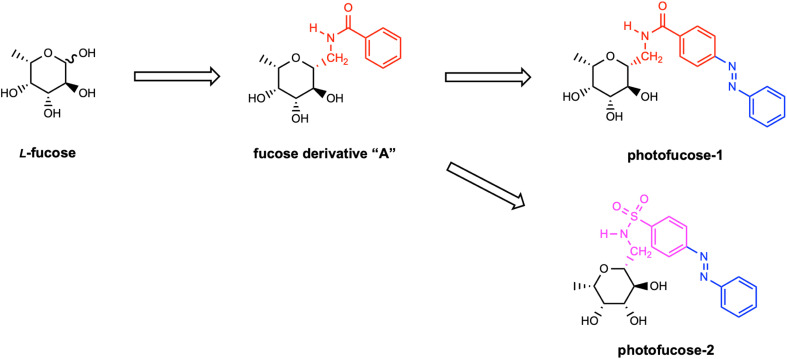
Design strategy for photofucose-1 and photofucose-2.

The design of the compounds in this study was inspired by ligands previously reported by Sommer *et al.*^10,39–41^ In their work, a series of derivatives were synthesized as potential LecB inhibitors, featuring a sugar moiety with aromatic extensions. They generated a diverse set of candidates by varying the nature of the sugar, the aromatic group and the linker connecting them. As previously discussed, fucosides exhibit higher affinities for LecB than mannosides, making them ideal scaffolds for the development of potent inhibitors. Accordingly, we designed novel inhibitors featuring a fucose-containing core conjugated to a benzene-modified moiety. For the first candidate, photofucose-1, we started from the structure of compound “A” from Scheme 1 and introduced an azobenzene extension to confer photo-switching features. Azobenzene was chosen among various photoswitches due to its well-documented advantages, including a straightforward synthetic route, structural simplicity, stability under biological conditions, and high efficiency in reversible photochemical isomerization.

Building on previous research by Sommer *et al.*,^10,12,39,40^ which demonstrated that sulfonamides exhibit stronger inhibitory effects than carboxamides, we further refined our design strategy. To enhance binding affinity and improve the overall pharmacophoric profile, we developed the second candidate, photofucose-2, by modifying the linker between the sugar and the azobenzene moiety, replacing the carboxamide with a sulfonyl amide group. This structural modification was aimed at optimizing interactions with LecB, exploiting the superior hydrogen-bonding and electronic properties of sulfonamides compared to their carboxamide counterparts.

### 2.2 Chemical synthesis & characterization

**Scheme 2:**
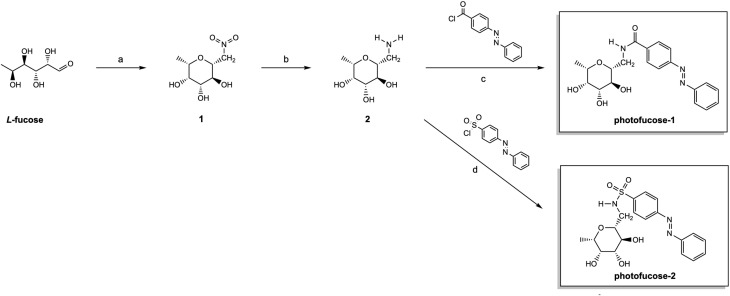
Synthesis of photofucose-1 and photofucose-2. (a) MeNO_2_, DBU, molecular sieves 3 Å, 1,4-dioxane, 50 °C, 1.5 d; (b) Pt/C, H_2_, MeOH, rt, 2 d; (c and d) acyl/sulfonyl chloride, Et_3_N, DMF, 0 °C, 3 d.

The synthesis of photofucose-1 and photofucose-2 followed previously reported pathways,^39–41^ starting from the key intermediate 2 (Scheme 2). Precursor 1 was obtained *via* a Henry condensation reaction using 1,8-diazabicyclo[5.4.0]undec-7-ene (DBU) as a catalyst on commercially available l-fucose in 1,4-dioxane and subsequently heated at 50 °C.^10,42^ Since the reaction yield was unsatisfactory to us, various reaction conditions were tested, with the highest yield achieved by adding DBU to a pre-heated (50 °C) reaction mixture (Table 1). Subsequent catalytic hydrogenation under H_2_ atmosphere, Pt/C in methanol efficiently reduced 1 to yield the desired intermediate 2. Final compounds photofucose-1 and photofucose-2 were obtained through a coupling reaction of 2 with the respective photoswitchable moieties, both commercially available, using the same general procedure for amide and sulfonamide coupling described by Sommer *et al.*^10,39^

**Table 1:** Reaction conditions tested for the Henry condensation reaction

Reagents	Test 1	Test 2	Test 3	Test 4	Test 5	Test 6
Eq. DBU	1.20	1.20	1.20	1.20	1.20	1.20
Eq. CH_3_NO_2_	32.00	32.00	16.00	10.00	16.00	16.00
Reaction temperature before DBU addition (°C)	25	25	25	25	50	50
Reaction time (h)	26	26	20	23.5	24.5	26.5
Yield	*24*.*60%*	*9*.*35%*	*12*.*09%*	*10*.*89%*	***51*.*12%***	***40*.*36%***

The purity of photofucose-1 and photofucose-2 was confirmed by UPLC-MS (99.99%), which also identified two distinct peaks corresponding to the *trans* and *cis* isomers. Under benchtop conditions, photofucose-1 was 90% *trans* and 10% *cis*, while photofucose-2 was 92% *trans* and 8% *cis* (Fig. S1). Stability testing under identical analytical conditions confirmed that photofucose-1 and photofucose-2 remain intact over time. Weekly UPLC-MS analysis for over two months showed no new peaks, indicating no detectable degradation (Fig. S2).

### 2.3 Photochemical characterization

**Fig. 1:**
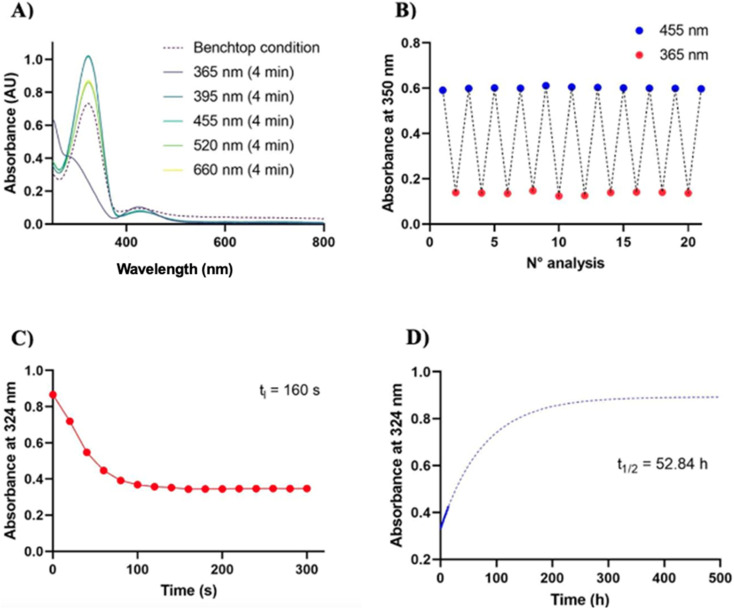
Photochromic characterization of photofucose-1. (A) Wavelength selection. UV-vis spectrophotometer analysis displays that 365 nm is the suitable wavelength for the *trans* → *cis* isomerization, and 395 nm for the reverse isomerization. To select the suitable wavelengths for the isomerization, samples were irradiated using LEDs of ThorLabs^®^, Herolab^®^, RoHS^®^, Vilber^®^ and Lepro^®^ at 365, 385–400, 470, 525 and 630 nm, emitting the monochromatic wavelengths. Intensity is reported in SI (Table S1) (B) photofatigue. The compound does not show any sign of fatigue in the timeframe tested; the transition is reversible and can be repeated several times without any noticeable loss of absorption. Cycle of irradiation: 3 minutes at 365 nm followed by 3 minutes at 455 nm. (C) Irradiation time. Photostationary state at 365 nm is reached after 160 seconds irradiation. Measures were performed at a 100 mm distance from the LED along the emission axis. (D) Relaxation time. UV-vis absorption studies show robust photochromic behavior in aqueous solution. The derivative follows a slow kinetics with a cis isomer half-life extended up to 52.84 h at 37 °C, which was estimated using the one phase association function in GraphPad^®^ Prism 10.5.0 software (nonlinear regression curve fitting), measuring absorbance at 324 nm.

**Fig. 2:**
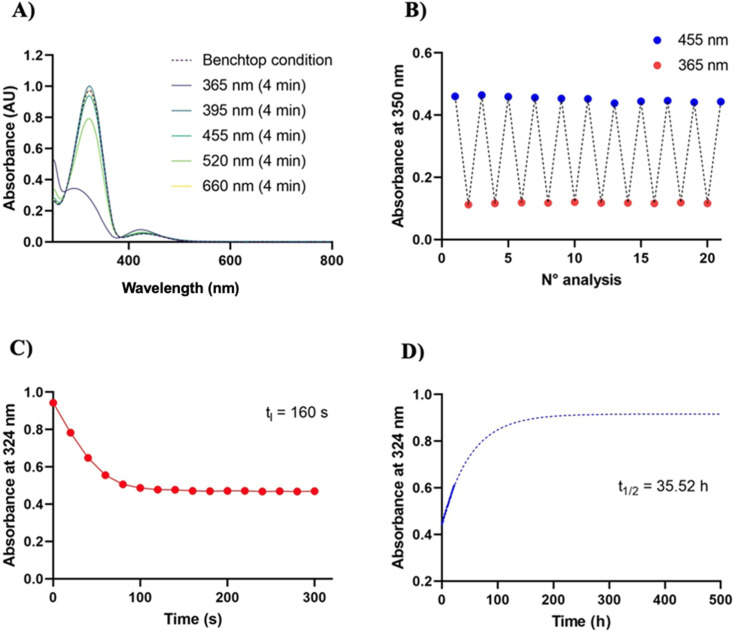
Photochromic characterization of photofucose-2. (A) Wavelength selection. UV-vis spectrophotometer analysis shows that 365 nm is the optimal wavelength for the *trans* → *cis* isomerization, while 395 nm is suitable for the reverse isomerization. Measures were performed at a 100 mm distance from the LED along the emission axis. To select the suitable wavelengths for the isomerization, samples were irradiated using LEDs of ThorLabs^®^, Herolab^®^, RoHS^®^, Vilber^®^ and Lepro^®^ at 365, 385–400, 470, 525 and 630 nm, emitting the monochromatic wavelengths. Intensity is reported in SI (Table S1) (B) photofatigue. The derivative shows no sign of fatigue in the timeframe tested, with the transition remaining highly reversible and repeatable without any noticeable loss of absorption after multiple cycles. Cycle of irradiation: 3 minutes at 365 nm followed by 3 minutes at 455 nm. (C) Irradiation time. Photostationary state at 365 nm is achieved after 160 seconds irradiation. (D) Relaxation time. UV-vis absorption measurements confirm a robust photochromic behavior in aqueous solution. The compound exhibits slow relaxation kinetics, with the *cis* isomer half-life estimated at 35.52 h at 37 °C. This value was estimated using the one phase association function in GraphPad^®^ Prism 10.5.0 software (nonlinear regression curve fitting), based on absorbance measurements at 324 nm.

A key requirement for a molecule to be used in photopharmacology is its ability to respond effectively and reversibly to light. This involves rapid toggling between two configurations through photoisomerization, with a relatively high *trans*/*cis* photoconversion ratio. As expected, photofucose-1 and photofucose-2 predominantly exist in their *trans* conformation in the dark at room temperature. Switching between the two isomers was achieved under proper illumination. Photofucose-1 and photofucose-2 were investigated through UV-vis absorption spectroscopy in DMSO and PBS buffer, as well as UPLC-MS in DMSO and ACN (Fig. 1, 2, S3, S4, Tables 2 and 3). The photoisomerization behavior of the two derivatives was investigated using absorption spectroscopy at selected wavelengths (365 nm, 395 nm, 455 nm, 520 nm, and 660 nm), with samples irradiated for 4 minutes at each wavelength. Both compounds underwent *trans*-to-*cis* photoisomerization upon UV light exposure (365 nm), while blue light (395 nm) effectively induced the reverse *cis*-to-*trans* isomerization. In the absence of light, the *cis* isomer spontaneously reverted to the *trans* form in the dark, requiring over 24 hours for complete relaxation.

**Table 2:** Proportion of *cis* and *trans* isomers of photofucose-1 as determined by UPLC-MS after illumination at different wavelengths

Wavelength	% *cis*	% *trans*
Benchtop	9.98	90.02
365 nm	**66.36**	33.64
385–400 nm	9.39	**90.61**
470 nm	19.79	80.21

**Table 3:** Proportion of *cis* and *trans* isomers of photofucose-2 as determined by UPLC-MS after illumination at different wavelengths

Wavelength	% *cis*	% *trans*
Benchtop	7.56	92.44
365 nm	**48.22**	51.78
385–400 nm	7.15	**92.85**
470 nm	16.35	83.65

To assess resistance to photofatigue, the compounds were subjected to 10 irradiation cycles (3 minutes at 365 nm followed by 3 minutes at 455 nm). No significant material degradation or loss of absorbance was observed, demonstrating the reversibility and stability of the photoisomerization process even after repeated switching. The thermal stability of the *cis* isomers was evaluated in DMSO and in PBS buffer (pH 7.3 to 7.5) at 20 °C and 37 °C in the dark. Both compounds exhibited a half-life exceeding 24 hours at 37 °C, with photofucose-1 showing greater stability in its *cis* form compared to photofucose-2. As expected, the *cis* isomers of both compounds displayed longer half-lives at 20 °C than at 37 °C. The kinetics of photoisomerization were investigated by irradiation at 365 nm for 5 minutes, with measurements taken every 20 seconds. Both compounds required approximately 160 seconds to reach their photostationary state (PSS). UPLC-MS analysis confirmed the *cis*/*trans* ratio at PSS after 5 minutes of illumination at 365 nm, 385–400 nm, and 470 nm. Although the quantum yields of the *trans*-to-*cis* photoreactions were not complete, a substantial fraction of the *cis* isomer was achieved. Upon 365 nm illumination, the *cis* fraction of photofucose-1 increased from 9.98% in the dark-adapted state to 66.36%, while photofucose-2 shifted from 7.56% to 48.22%. Maximum *trans* enrichment was observed at 385–400 nm.

## 3 LecB production

The sequence of *PA* LecB was cloned with an N-terminal 6xHis tag into a pEX-N-His vector (Addgene) conferring ampicillin resistance. Expression was carried out in *Escherichia coli* BL21(DE3) cells (Invitrogen) induced with 0.1 mM IPTG and grown at 18 °C for 16 h.

Following induction, cells were harvested, lysed, and the lysate clarified by centrifugation. The His-tagged LecB was purified by Ni^2+^-affinity chromatography. To remove the imidazole present in the elution buffer from the Nickel affinity chromatography step, a buffer exchange column (PD-10, Cytiva) was employed, resulting in pure protein. The final yield of pure protein was determined to be 2.5 mg per liter of culture.

## 4 MALDI-TOF analysis

The His-tagged LecB protein was characterized using MALDI-TOF analysis to confirm that its molecular weight matched the expected value. Fig. S15 shows the His-tagged LecB monomer (approximately 14.187 kDa), along with its dimer and a subunit with a molecular weight approximately half that of the monomer.

## 5 Isothermal titration calorimetry (ITC)

**Fig. 3:**
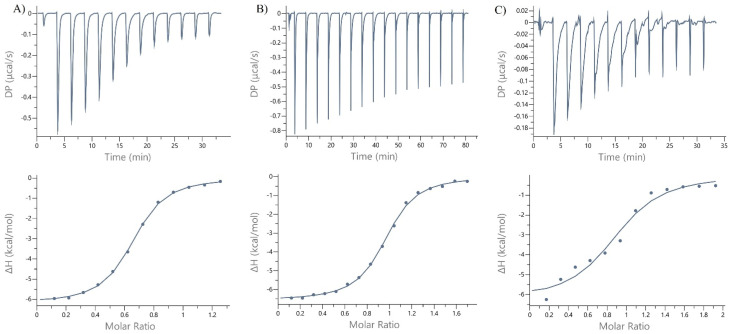
ITC measurements of l-fucose (A), photofucose-2 (B) and photofucose-2 after 5 minutes of illumination at 365 nm (C). Measurements were carried out at a protein concentration of 80 µM and a ligand concentration of 800 µM. All measurements were performed in triplicate (*n* = 3).

ITC experiments were performed using a Microcal PEAQ-ITC (Malvern Panalytical). Experiments were conducted to investigate the interaction of LecB with a range of small molecules (l-fucose, *trans*-photofucose-2 and *cis*-photofucose-2) after 5 minutes of illumination at 365 nm. All tested molecules demonstrated the ability to interact with LecB, as shown in Fig. 3. Photofucose-1 exhibited solubility issues under the conditions used in the analysis and was therefore abandoned. Remarkably, *trans*-photofucose-2 displayed a similar binding affinity to l-fucose (Table 4) as indicated by the similar *K*_d_ value. Interestingly, *trans*-photofucose-2 exhibited a slightly lower *K*_d_ than *cis*-photofucose-2, suggesting binding affinity modulation associated with photoisomerization.

**Table 4:** ITC measurements results. All measurements were performed in triplicate (*n* = 3)

Ligand	*K*_d_ [µM]	*N* (sites)	−Δ*G* [kcal mol^−1^]	−Δ*H* [kcal mol^−1^]	*T*Δ*S* [kJ mol^−1^]
l-Fucose	1.710 ± 0.293	0.770 ± 0.243	−7.870 ± 0.098	−6.563 ± 0.675	−1.049 ± 0.878
*trans*-photofucose-2	1.820 ± 0.408	1.006 ± 0.059	−7.843 ± 0.124	−7.237 ± 0.787	−0.681 ± 0.372
*cis*-photofucose-2	4.713 ± 2.606	0.851 ± 0.177	−7.340 ± 0.284	−8.47 ± 0.212	−1.24 ± 0.060

## 6 Crystal structure analysis

We determined the crystal structure of LecB in its ligand-bound state at 1.49 Å from crystals soaked with *trans*-photofucose-2. The structure was solved by molecular replacement using the structure of LecB from *Pseudomonas aeruginosa* strain 14 (PDB ID: 5MAY) as the search model.^41^

The final structure, which is tetrameric, was refined to *R*/*R*_free_ factors of 0.16/0.22 (see Table 5). The final coordinates were deposited in the Protein Data Bank (PDB) under accession code 9HD4.

**Table 5:** X-ray data collection and refinement statistics. Statistics for the highest-resolution shell are shown in parentheses

LecB-photofucose-2 complex (9HD4)	
Wavelength (Å)	0.95374
Resolution range (Å)	50.95–1.491 (1.544–1.491)
Space group	*C* 2 2 2_1_
Unit cell (Å, °)	64.21, 83.72, 158.88, 90.0, 90.0, 90.0
Total reflections	139 078 (13 867)
Unique reflections	69 660 (6934)
Multiplicity	2.0 (2.0)
Completeness (%)	99.38 (99.80)
Mean I/sigma (I)	8.19 (1.34)
Wilson *B*-factor	12.17
R-merge	0.05888 (0.559)
R-meas	0.08327 (0.7905)
R-pim	0.05888 (0.559)
CC1/2	0.996 (0.463)
CC*	0.999 (0.796)
Reflections used in refinement	69 644 (6933)
Reflections used for R-free	3511 (343)
R-work	0.1585 (0.3050)
R-free	0.2205 (0.3537)
CC (work)	0.908 (0.623)
CC (free)	0.952 (0.603)
Number of non-hydrogen atoms	3942
Macromolecules	3418
Ligands	126
Solvent	398
Protein residues	456
RMS (bonds) (Å)	0.012
RMS (angles) (°)	1.76
Ramachandran favored (%)	97.29
Ramachandran allowed (%)	2.71
Ramachandran outliers (%)	0.00
Rotamer outliers (%)	1.57
Clashscore	3.62
Average *B*-factor	19.08
Macromolecules	16.30
Ligands	57.85
Solvent	30.73

Consistent with previous LecB structures, the protein adopts a tetrameric assembly in which each monomer contributes a canonical fucose-binding site containing two tightly bound Ca^2+^ ions.^43^ The ligand is bound to the protein at the sugar-binding site *via* its fucose moiety, and this interaction is consistently observed in all four chains of the LecB tetramer. The three hydroxyl groups of the fucose moiety coordinate two calcium ions, thereby forming the core of the recognition network that stabilizes the ligand–protein complex. These calcium ions bridge the sugar hydroxyls to a network of acidic and polar residues, including the residues Asn21, Glu95, Asp99, Asp101, Asn103 and Asp104, which have been shown to be essential for carbohydrate recognition in LecB.^41^ The superimposition of the fucose binding with reference structure 5MAY is shown in Fig. S19.

Overall, the Ca^2+^-dependent structural organization is consistent with LecB's function in biofilm formation, in which cooperative carbohydrate binding enhances matrix cohesion.^44^

**Fig. 4:**
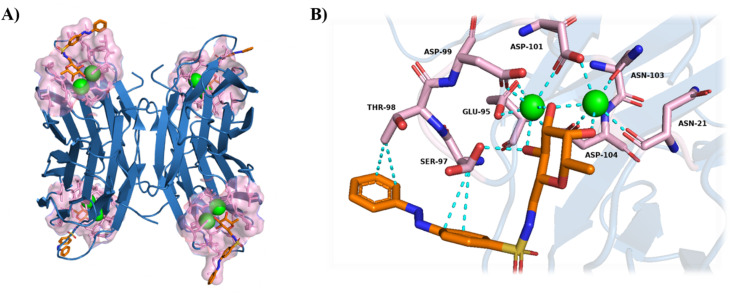
(A) Tetrameric structure of LecB in complex with photofucose-2 (PDB: 9HD4) (green spheres: calcium ions, red: oxygens, blue: nitrogen, pink: binding pocket, orange: photofucose-2) (B) detailed view of the LecB binding site showing the key residues that interact with *trans*-photofucose-2.

In our structure, an additional H-bonding interaction is established between one of the hydroxyl groups of l-fucose and Ser97, further contributing to the stabilization of the complex (Fig. 4).

**Fig. 5:**
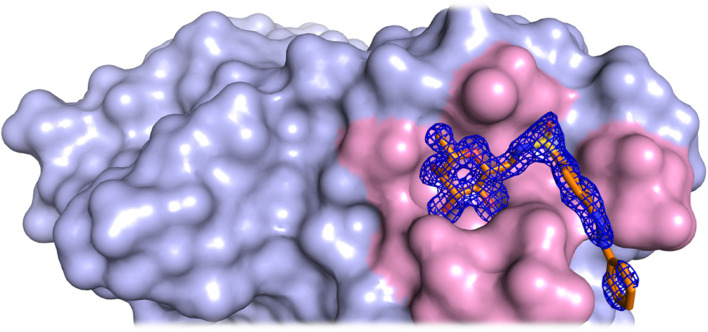
Binding site of LecB (shown in pink color) in complex with the *trans*-photofucose-2 ligand. The electron density (2Fo-Fc) map of *trans*-photofucose-2 is shown at 1.0 sigma level.

In contrast to the fucose moiety, the electron density of the azobenzene moiety is less well-defined, likely due to its inherent conformational flexibility. The extent by which this electron density is visible varies among the four chains of the homotetramer. Notably, the *trans*-photofucose-2 conformation is best defined in chain B of the tetramer. This can be attributed to the orientation of the azobenzene group, which forms stabilizing (≤3.5 Å) van der Waals interactions with residues Ser97 and Thr98 of the protein (Fig. 4B and 5).

## 7 Experimental section

### 7.1 General materials and methods

Chemicals and solvents used are commercially available with proper purity levels, checked by the suppliers. Reactions were monitored by thin layer chromatography (TLC) analyses performed with commercial silica gel 60 F254 aluminium sheets (Merck^®^). Spots were observed with UV-lamp under 254 and/or 365 nm and stained with potassium permanganate or ninhydrin reagent if needed. ^1^H-NMR and ^13^C -NMR spectra were recorded on a Varian Mercury-300 instrument (300 MHz and 75 MHz respectively) and on a Bruker Avance-400 instrument (400 MHz and 100 MHz respectively). Chemical shifts (*δ*) are reported in parts per million (ppm) and are relative to the peak of the internal standard tetramethylsilane (*δ* = 0.00 ppm) using the signal of the residual non-deuterated solvent [CDCl_3_*δ* = 7.26 ppm (^1^H), *δ* = 77.00 ppm (^13^C), Methanol-d_4_*δ* = 3.31 ppm (^1^H), = 49.00 ppm (^13^C), DMSO-d_6_*δ* = 2.50 ppm (^1^H), = 39.52 ppm (^13^C)]. Processing of the spectra was performed with MestReNova^®^ 8.1.1. Coupling constants (*J*) are reported in Hertz (Hz). For each compound analysed, the instrument and the specific deuterated solvent used is reported (Fig. S5–S12). Samples were analysed using an UPLC Waters Acquity, column: Acquity UPLC BEH-C18, 1.7 µm, 2.1 mm × 50 mm, column temperature (30.0 °C ± 5.0), gradient: from 0.01% TFA in water/MeCN 90%/10–5%/95%; flow: 0.500 mL min^−1^; time: 8 min; detector: PDA, 220–320 nm, SQ detector with an electrospray ion source (ESI/APCI). Samples were prepared at the concentration of 100 µM in ACN (HPLC grade) and illuminated with wavelength of 365 nm, 385–400 nm and 470 nm for 5 minutes. Samples were also analysed using a Waters ACQUITY QSM detector equipped with an electrospray ionization (ESI) interface. Capillary, kV: 0.7 and cone, V: 40. LC 59. Column: acquity UPLC BEH C18 2.1 × 50 mm, 1.7 µm. Solvent A: formic acid and solvent B: acetonitrile. Spectra have been scanned between 0 and 900 Da with values every 0.1 seconds and peaks are given as mass/charge (*m*/*z*) ratio. For photofucose-1 and photofucose-2 high-resolution molecular masses (HRMS) were determined on a Waters Synapt G2-Si hybrid quadrupole time-of-flight (TOF) mass spectrometer equipped with an electron spray ion source (ESI) (Fig. S13 and S14).

### 7.2 General procedure for Henry condensation with 4,6-*O*-benzylidenylated and non-protected l-fucose*via* DBU-catalysis (intermediate 1)

DBU (1.1 mL, 7.3 mmol) was slowly added to a mixture of l-fucose (1.0 g, 6.1 mmol), MeNO_2_ (10 mL, 190 mmol) and molecular sieves (3 Å, 5 g) in 1,4-dioxane (25 mL) at 50 °C. The slurry was stirred under N_2_ at 50 °C for 25 h and filtered. The filtrate was concentrated *in vacuo*. The residual oil was chromatographed (SiO_2_, EtOAc/MeOH 9/1). The obtained crystals were recrystallized from THF/(iPr)_2_O to afford the desired product GTZ1 as a white powder (51%). Melting point: 182 °C. *R*_f_ = 0.41 (ethyl acetate/methanol 9 : 1). ^1^H NMR (300 MHz, CD_3_OD) *δ* 4.80 (d, *J* = 2.3 Hz, 1H), 4.48 (dd, *J* = 13.1, 9.6 Hz, 1H), 3.89 (td, *J* = 9.5, 2.3 Hz, 1H), 3.67–3.56 (m, 2H), 3.49 (dd, *J* = 9.3, 3.1 Hz, 1H), 3.42 (t, *J* = 9.3 Hz, 1H), 1.20 (d, *J* = 6.5 Hz, 3H). ^13^C NMR (75 MHz, DMSO-d_6_) *δ* 75.49, 74.99, 73.07, 70.98, 68.28, 65.32, 20.47.

### 7.3 General procedure for the reduction of ß-l-fucopyranosyl methylamine (intermediate 2)

A suspension of intermediate 1 (1.0 g, 4.83 mmol) and 5% Pt–C (111 mg, 0.03 mmol) in CH_3_OH (43.0 mL) was stirred under a H_2_ atmosphere at room temperature for 72 h. The resulting mixture was filtered over Celite®, and the solvent was evaporated under reduced pressure to give ß-l-fucopyranosyl methylamine (82%) as a white solid. The obtained compound was used in the next step without further purification. *R*_f_ = 0.1 (dichloromethane/methanol 7 : 3). ^1^H NMR (300 MHz, CD_3_OD) *δ* 3.64–3.56 (m, 2H), 3.43 (dd, *J* = 5.3, 2.3 Hz, 2H), 3.34 (s, 1H), 2.97 (dd, *J* = 13.3, 3.0 Hz, 1H), 2.73 (dd, *J* = 13.4, 7.2 Hz, 1H), 1.24 (d, *J* = 6.4 Hz, 3H). ^13^C NMR (75 MHz, DMSO-d_6_) *δ* 76.73, 74.87 (2C), 74.45, 71.85, 68.58, 17.33.

### 7.4 General procedure for amide and sulfonamide couplings

#### 7.4.1 4-((*E*)-Phenyldiazenyl)-*N*-(((2*R*,3*S*,4*R*,5*S*,6*S*)-3,4,5-trihydroxy-6-methyltetrahydro-2*H*-pyran-2-yl)methyl)benzamide (photofucose-1)

ß-l-Fucopyranosyl methylamine (55.6 mg, 0.313 mmol) and triethylamine (47.6 mg, 0.47 mmol) were dissolved in anhydrous DMF (1.7 mL) and cooled to 0 °C. The corresponding acyl chloride (92.13 mg, 0.377 mmol) dissolved in DMF (4.70 mL) was added dropwise under nitrogen atmosphere. The reaction was then stirred for further 72 h at room temperature. Saturated aqueous NH_4_Cl was added, and the mixture was extracted with EtOAc. The combined organic layers were dried over Na_2_SO_4_, filtered, and concentrated *in vacuo*. The residue was purified by chromatography on silica (CH_2_Cl_2_ to CH_2_Cl_2_/EtOH = 10 : 1 or CH_2_Cl_2_/MeOH = 10 : 1). The purification provided photofucose-1 (36%, mixture of *cis* and *trans* isomers) as an orange powder. *R*_f_ = 0.216 (dichloromethane/methanol 9.5 : 0.5). Retention time: 2.094 min *cis* isomer and 2.674 min *trans* isomer. ^1^H NMR (300 MHz, CD_3_OD) *δ* 8.05–8.01 (m, 1H), 8.00 (d, *J* = 5.6 Hz, 3H), 7.95 (dd, *J* = 8.0, 1.9 Hz, 2H), 7.61–7.50 (m, 3H), 3.75–3.71 (m, 2H), 3.68–3.63 (m, 2H), 3.51 (dd, *J* = 5.4, 2.5 Hz, 2H), 3.34 (s, 1H), 1.26 (d, *J* = 6.4 Hz, 3H). ^13^C NMR (75 MHz, DMSO-d_6_) *δ* 166.27, 153.65, 152.34, 137.08, 132.45, 130.00, 129.10, 123.18, 122.77, 78.83, 74.96, 74.20, 72.01, 71.90, 69.22, 69.10, 42.23, 17.57.

#### 7.4.2 4-((*E*)-phenyldiazenyl)-*N*-(((2*R*,3*S*,4*R*,5*S*,6*S*)-3,4,5-trihydroxy-6-methyltetrahydro-2*H*-pyran-2-yl)methyl)benzenesulfonamide (photofucose-2)

ß-l-Fucopyranosyl methylamine (100 mg, 0.564 mmol) and triethylamine (86.66 mg, 0.847 mmol) were dissolved in anhydrous DMF (3 mL) and cooled to 0 °C. The corresponding sulphonyl chloride (190 mg, 0.677 mmol) dissolved in DMF (8.40 mL) was added dropwise under nitrogen atmosphere. The reaction was allowed to warm to room temperature and was stirred for further 36 h. Saturated aqueous NH_4_Cl was added and extracted with EtOAc. The combined organic layers were dried over Na_2_SO_4_, filtered, and concentrated *in vacuo*. The residue was purified by chromatography on silica (CH_2_Cl_2_ to CH_2_Cl_2_/EtOH = 10 : 1 or CH_2_Cl_2_/MeOH = 10 : 1) and after with crystallization with EtOAc. The purification provided photofucose-2 (15%, mixture of *cis* and *trans* isomers) as a red powder. *R*_f_ = 0.576 (ethyl acetate/methanol 9 : 1). Retention time: 2.201 *cis* isomer and 2.783 *trans* isomer. ^1^H NMR 1H NMR (400 MHz, DMSO-d_6_) *δ* 8.07–7.99 (m, 4H), 7.97–7.91 (m, 2H), 7.77 (t, *J* = 5.9 Hz, 1H, NH), 7.64 (dd, *J* = 5.1, 2.0 Hz, 3H), 4.79 (d, *J* = 5.2 Hz, 1H), 4.58 (d, *J* = 5.7 Hz, 1H), 4.25 (d, *J* = 5.5 Hz, 1H), 3.38 (t, *J* = 4.4 Hz, 2H), 3.26 (d, *J* = 8.2 Hz, 1H), 3.23–3.10 (m, 2H), 3.04–2.97 (m, 1H), 2.77 (t, *J* = 13.6 Hz, 1H), 1.04 (d, *J* = 6.4 Hz, 3H).

### 7.5 Optical instruments, protocols and methods

The photochromic characterization of the compounds was performed using two different instruments. A Microplate Spectrophotometer Multiskan FC® (Thermo Scientific), equipped with an incubator and quartz cuvettes (10 mm light path), was used for the photochemical characterization and photofatigue analysis. A NanoDrop OneC® (Thermo Scientific), using PMMA cuvettes (10 mm light path), was employed to assess relaxion time and time-dependent behavior. To achieve photoconversion, 1.5 mL of the solution in a cuvette was irradiated inside a closed box for 3–5 minutes using LED arrays at the selected wavelength.

Wavelength selection: to identify suitable wavelengths for isomerization, samples were irradiated using LEDs from ThorLabs®, Herolab®, RoHS®, Vilber® and Lepro®, emitting monochromatic light at 365, 385–400, 470, 525 and 630 nm. The corresponding light intensities are reported in the SI (Table S1). To qualitatively assess compound conversion, the cuvette was subsequently placed in a UV-vis spectrophotometer. Measures were performed at a distance of 100 mm from the LED source along the emission axis.

UV-vis spectra: UV-vis spectra for the photochemical characterization of the compounds were recorded using standard quartz cuvettes with a 10 mm optical path, following 3 minutes of irradiation. Samples were prepared at a concentration of 50 µM in PBS buffer containing 0.5% DMSO.

Photofatigue resistance: to determine potential degradation after prolonged irradiation, the solution was subjected to 10 cycles of alternating light exposure: 3 minutes at 365 nm followed by 3 minutes at 455 nm. Absorbance values were checked at the wavelength with the biggest difference in absorption between the *cis* and the *trans* isomer. Samples were prepared at a concentration of 50 µM in 0.5% DMSO and PBS buffer.

Kinetics: thermal relaxation was measured by switching the photochromic compounds into the thermodynamically less stable *trans*-photoisomer using 365 nm light for 5 minutes at two different temperatures. Then, relaxation was determined by monitoring the change of absorption at the indicated wavelength at room temperature (20 °C) and human body temperature (37 °C). Samples were prepared at a concentration of 50 µM in 0.5% DMSO and PBS buffer. Half-lives were determined by nonlinear regression (curve fit) – exponential – plateau followed by one-phase decay model with Graphpad^®^ Prism 10.5.0. software.

*Cis*/*trans* ratios measurements: the photostationary distribution (PSD) of the photostationary states (PSS) and after irradiation with different wavelengths were determined by Acquity UPLC BEH-C18. Samples were prepared at a concentration of 100 µM in 1.00% DMSO and ACN and irradiated for 5 minutes.

Time dependence: to estimate the time required for the photo conversion the cuvette with the solution of photofucose-1 or photofucose-2 was irradiated every 20 seconds at the wavelengths of 365 nm until variations in the absorbance values were no longer observed. Absorbance was measured at the wavelength showing the greatest difference in absorption between the *cis* and the *trans* isomers, the same wavelength used for the kinetics analysis. Samples were prepared at a concentration of 50 µM in 0.5% DMSO and PBS buffer.

### 7.6 Cloning, expression, and purification of *Pseudomonas aeruginosa* LecB

The *PA* LecB gene was cloned into Pxp vector with an N-terminal His-tag for purification. Primers designed using SnapGene included overhangs for SLIC cloning. The recombinant plasmid was verified by gel electrophoresis, amplified by PCR, transformed into T7 cells (New England Biolabs), and subsequently extracted using a plasmid miniprep kit (Thermo Scientific) before being sent for sequencing (Azenta).

After the confirmation of the correct sequence, a single colony was picked up from a freshly transformed plate and inoculated into 50 mL of overnight culture supplemented with ampicillin at a concentration of 100 mg L^−1^. For the large scale production, 50 mL of overnight culture were inoculated into 4 L of Luria–Bertani (LB) broth supplemented with ampicillin at a concentration of 100 mg L^−1^ and grown at 37 °C until reaching an OD_600_ = 0.6. Protein expression was induced by supplementing the culture with 1-thio-β-d-galactopyranoside (IPTG) at final concentration of 0.1 mM, followed by an overnight incubation at 18 °C with shaking at 200 rpm. Cells were harvested using centrifugation and resuspended in lysis buffer (20 mM Tris–HCl pH 7.5, 100 mM NaCl, 100 µM CaCl_2_, imidazole 20 mM) supplemented with 0.2 mM protease phenylmethylsulfonyl fluoride (PMSF), 5 µL DNAse (ThermoFisher scientific). The cells were lysed by sonication on ice, and fraction was collected and filtered through a 0.45-micron filter. The filtered solution was loaded onto a 5 mL a HisTrap HP 5 mL (Cytiva) column pre-equilibrated with binding buffer (20 mM Tris–HCl pH 7.5, 100 mM NaCl, 100 µM CaCl_2_, 400 mM imidazole). The column was washed with 10 times column volumes of binding buffer and the samples were eluted with 10 column volumes of elution buffer (20 mM Tris–HCl pH 7.5, 100 mM NaCl, 100 µM CaCl_2_, 400 mM imidazole) using a gradient. The best faction was collected and subjected to buffer exchange using a PD-10 column (Cytiva) pre-equilibrated with dilution buffer (20 mM Tris–HCl pH 7.5, 100 mM NaCl, 100 µM CaCl_2_). Fractions containing the desired protein were collected, and their concentrations were measured using a NanoDrop spectrophotometer (Thermo Scientific). The samples were flash frozen in liquid nitrogen, and their purity was assessed by 12% SDS-PAGE.

### 7.7 MALDI-TOF analysis

Measurements were performed using a FLEX-PC autoflex TOF/TOF instrument (Bruker). Data were acquired in linear mode with positive polarity, and each spectrum was collected with 500 laser shots. Protein samples (1 µL, 10 mg mL^−1^) were acidified by mixing with 0.5 µL of formic acid. A drop of the resulting mixture was then combined with the matrix SA (sinapinic acid) and allowed to dry prior to data acquisition.

### 7.8 Isothermal titration calorimetry (ITC)

Measurements were performed with a Microcal PEAQ-ITC (Malvern Panalytical). The experiments were carried out in triplicate at 298.15 K. LecB and compounds were dissolved in 20 mM Tris pH 7.5, 100 mM NaCl, and 100 µM CaCl_2_. LecB was used at 80 µM concentration. A total of 13 injections of 3 µl of compound solutions at 800 µM were added at intervals of 150 s while stirring at 750 rpm. The experimental data were fitted to a theoretical titration curve using software supplied by Microcal. Calculations were performed using the standard equation:Δ*G* = Δ*H* − *T*Δ*S* = −*RT* ln *K*_a_where Δ*G*, Δ*H*, and *S* are the changes in free energy, enthalpy, and entropy, respectively, of binding, *T* is the absolute temperature, *R* = 8.32 J mol^−1^ K^−1^ and *K*_a_ is the association constant. All experiments were performed with *c* values 100 < *c* < 200 (*c* = *K*_a_*M*, where *M* is the initial concentration of the macromolecule).

### 7.9 Protein crystallization

Crystals of the apo-protein were grown at room temperature by the vapor diffusion method. A 1 µL drop of a 10 mg mL^−1^ protein sample was mixed with 1 µL of a solution containing 20% PEG 8K, 0.25 M LiCl, 0.1 M sodium acetate, pH 4.3. Crystals appeared within 1–2 weeks and used for soaking experiments. Soaking solutions of *trans*-photofucose-2 and *cis*-photofucose-2 (UV illumination 10 minutes at 365 nm wavelength) were prepared at 10 mM concentrations in the same buffer that afforded apo-crystals. Crystals were soaked for 2 hours in dark and then frozen in a chemically identical solution supplemented with 25% (v/v) glycerol prior to X-ray diffraction data collection.

### 7.10 X-ray data collection

Diffraction data were obtained using an Eiger2 XE 16 M detector and a radiation of *λ* = 0.9537 Å on the I04 beamline at the Diamond Light Source (Oxfordshire, United Kingdom). Data processing was done using the autoproc package.^45^ Data collection and refinement statistics are summarized in Table 5.

### 7.11 Structure determination and refinement

For structure determination, initial data were obtained *via* molecular replacement using Phaser, with the atomic coordinates of another LecB-ligand complex (PDB accession code: 5MAY) serving as the probe. Refinement was done using a combination of manual adjustments in Coot and iterative rounds of REFMAC5. Water molecules were added both manually and through automatic placement using the Coot_refine tool from the CCP4 package. The final crystallographic coordinates of the crystal structure are available in the RCSB (PDB accession code: 9HD4).

## 8 Conclusions

In this study, we designed, synthesized, and characterized two novel photoswitchable compounds, photofucose-1 and photofucose-2, as potential modulators of the lectin LecB from *Pseudomonas aeruginosa.* These compounds exhibit robust photochemical properties, significant thermal- and photo-stability, and the ability to undergo reversible photoisomerization between their *cis* and *trans* isomers. We thereby expressed and purified the target protein LecB and characterized the ligands structurally and biophysically. The ability of both molecules to interact with the protein was evaluated *via* ITC analysis. Our findings revealed that photofucose-2 displays a notable affinity for LecB, with a clear difference between its *cis* and *trans* conformations. Furthermore, we co-crystallized LecB with photofucose-2 in its *trans* conformation and solved the crystal structure of the complex at 1.49 Å resolution. This characterization allowed the elucidation of the specific binding mode of photofucose-2 to LecB, thus providing a structural framework for the future optimization of photoswitchable LecB inhibitors.

Our results demonstrate that light can be exploited as a remote, non-invasive trigger to modulate the activity of a bacterial lectin, offering a novel approach for targeting *PA* biofilms. The ability to switch between two ligand states in a controlled manner represents a promising strategy in antimicrobial research, potentially reducing the selective pressure associated with conventional antibiotics and mitigating the development of resistance. Although further investigations are necessary to optimize the pharmacokinetic and pharmacodynamic properties of these compounds, our findings lay a solid foundation for the development of a new class of light-responsive antimicrobial agents. Future work will focus on fine-tuning the photochromic properties of this scaffold, optimizing absorption and relaxation kinetics, and evaluating biological efficacy in more complex models of *PA* infections. Overall, this study represents a step forward in the field of photopharmacology and antimicrobial drug discovery, highlighting the potential of photoswitchable ligands as precision tools for combating bacterial virulence factors.

## Author contributions

SB, GT, AC and RC: methodology, investigation, formal analysis, writing and editing of the manuscript. CM, RC and EP: conceptualization and design of the research, funding acquisition, formal analysis, writing and editing of the manuscript. All authors reviewed and approved the final version of the manuscript.

## Conflicts of interest

There are no conflicts to declare.

## Supplementary Material

RA-015-D5RA06897E-s001

## Data Availability

The data supporting this article have been included as part of the supplementary information (SI). Crystallographic data for the LecB-photofucose-2 complex has been deposited at the PBD under accession number 9HD4. Supplementary information is available. See DOI: https://doi.org/10.1039/d5ra06897e.
