# Supplementary material for: Synthesis, photochemical and biological evaluation of novel photoswitchable glycomimetic ligands of Pseudomonas aeruginosa LecB
Source: RSC Adv. 2025 Dec 12;15(58):49796–808. doi: 10.1039/d5ra06897e (PMC12700220; doi:10.1039/d5ra06897e)
Supplement: RA-015-D5RA06897E-s001 [file RA-015-D5RA06897E-s001.pdf]

Supplementary Information (SI) for RSC Advances.
This journal is © The Royal Society of Chemistry 2025

SUPPLEMENTARY INFORMATION

Synthesis, Photochemical and Biological Evaluation of Novel Photoswitchable Glycomimetic Ligands of *Pseudomonas aeruginosa* LecB

Shapla Bhattacharya[1,2], Giorgia Tempra[3], Alessio Colleoni[3,4], Carlo Matera[3], Rossella Castagna[1,5]*, Emilio Parisini[1,6]*.

[1]Department of Biotechnology, Latvian Institute of Organic Synthesis, Aizkraukles 21, LV-1006, Riga, Latvia

[2]Faculty of Materials Science and Applied Chemistry, Riga Technical University, Paula Valdena 3, LV-1048, Riga, Latvia

[3]Department of Pharmaceutical Sciences, University of Milan, Via Luigi Mangiagalli 25, 20133 Milan, Italy

[4]Department of Chemistry, Biology and Biotechnology, University of Perugia, Via Elce di Sotto 8, 06123 Perugia, Italy

[5]Department of Chemistry, Materials and Chemical Engineering "G. Natta", Politecnico di Milano, Piazza Leonardo da Vinci 32, 20133 Milano, Italy

[6]Department of Chemistry "G. Ciamician", University of Bologna, Via P. Gobetti 85, 40129 Bologna, Italy

* To whom correspondence should be addressed: rossella.castagna@polimi.it emilio.parisini@osi.lv

Table of Contents:

1. Chemical characterization
2. Photochemical characterization
3. NMR spectra
4. Mass spectra
5. MALDI-TOF
6. Illumination protocols

1. Chemical characterization

The purity of **photofucose-1** and **photofucose-2** was assessed by ultra-performance liquid chromatography coupled with mass spectrometry (UPLC-MS) on a Waters Acquity column: Acquity UPLC BEH-C18, 1.7 µm, 2.1 mm x 50 mm, gradient: from 0.01% TFA in water/MeCN 90%/10% – 5%/95%; flow rate 0.5 ml/min; run time 8 min; detector PDA (photodiode matrix) 220-320 nm. Mass spectroscopy (MS) apparatus: Waters ACQUITY QSM detector equipped with an electrospray ion source (ESI/APCI). Samples were prepared in acetonitrile (ACN) from a 100 µM stock in dimethyl sulfoxide (DMSO). The purity of both molecules was found to be 99.99%. Additionally, the analysis revealed the presence of two distinct, well-separated peaks, corresponding to the *cis* and *trans* isomers of each compound. Under benchtop conditions, **photofucose-1** was obtained as 9.98% *cis* and 90.02% *trans*, while **photofucose-2** was obtained as 7.56% *cis* and 92.44% *trans*.

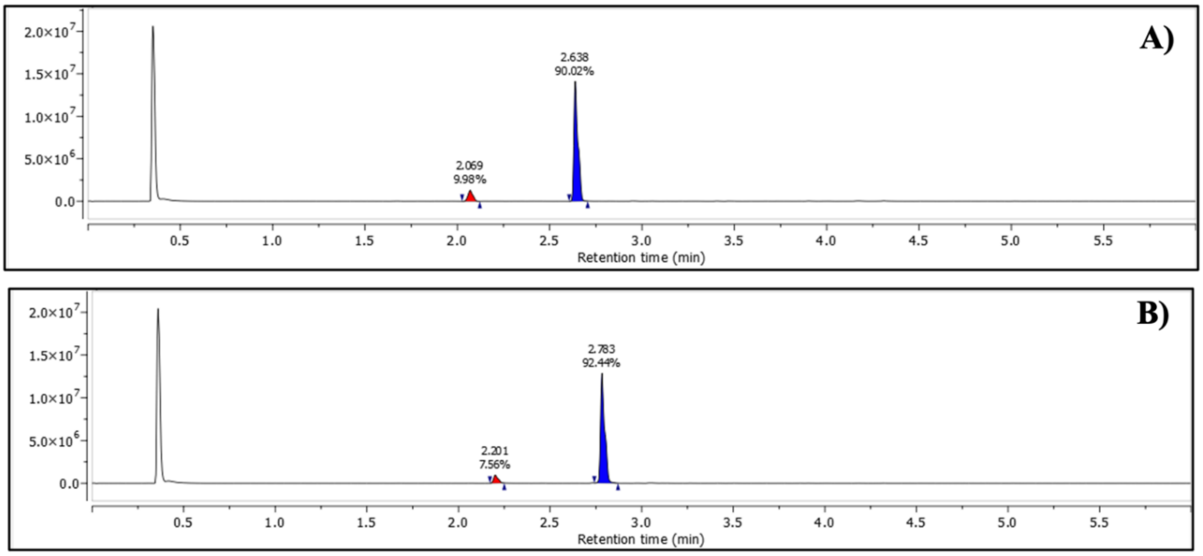

Figure S1. A) photofucose-1 and **B) photofucose-2** in their photostationary states as determined by UPLC-MS under benchtop conditions. The red peak corresponds to the *cis* isomer, while the blue peak corresponds to the *trans* isomer.

The stability test was conducted under identical analytical conditions, demonstrating that **photofucose-1** and **photofucose-2** remain stable over time and that the analysis can be repeated multiple times without any notable loss of compound integrity. To ascertain whether any degradation of the molecules had occurred, the same samples of **photofucose-1** and **photofucose-2** were subjected to UPLC-MS testing weekly for a period exceeding two months. As shown in Figures S2, no new peaks were observed over time, indicating that no degradation products had formed during the testing period.

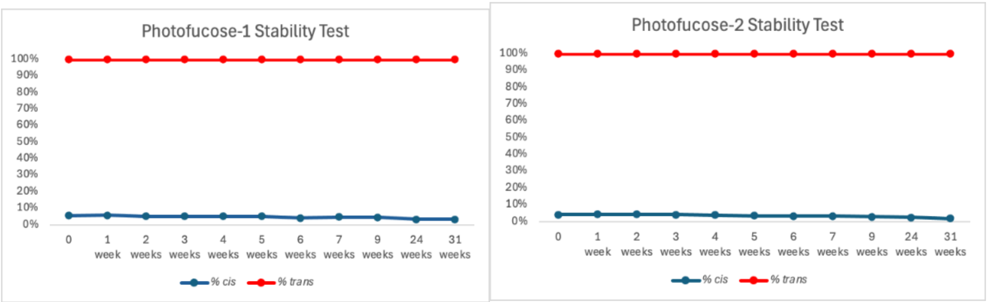

Figure S2. Stability test of **photofucose-1** (left) and **photofucose-2** (right) as determined by UPLC-MS under benchtop conditions. Samples were stored at 5°C in the dark.

2. Photochemical characterization

The photochemical properties of **photofucose-1** and **photofucose-2** were assessed by ultra-performance liquid chromatography coupled with mass spectrometry (UPLC-MS) on a Waters Acquity column: Acquity UPLC BEH-C18, 1.7 µm, 2.1 mm x 50 mm, gradient: from 0.01% TFA in water/MeCN 90%/10% – 5%/95%; flow rate 0.2 ml/min; run time 8 min; detector PDA (photodiode matrix) 220-320 nm. SQ detector with an electrospray ion source (ESI/APCI). Samples were prepared in dimethyl sulfoxide (DMSO) and acetonitrile (ACN).

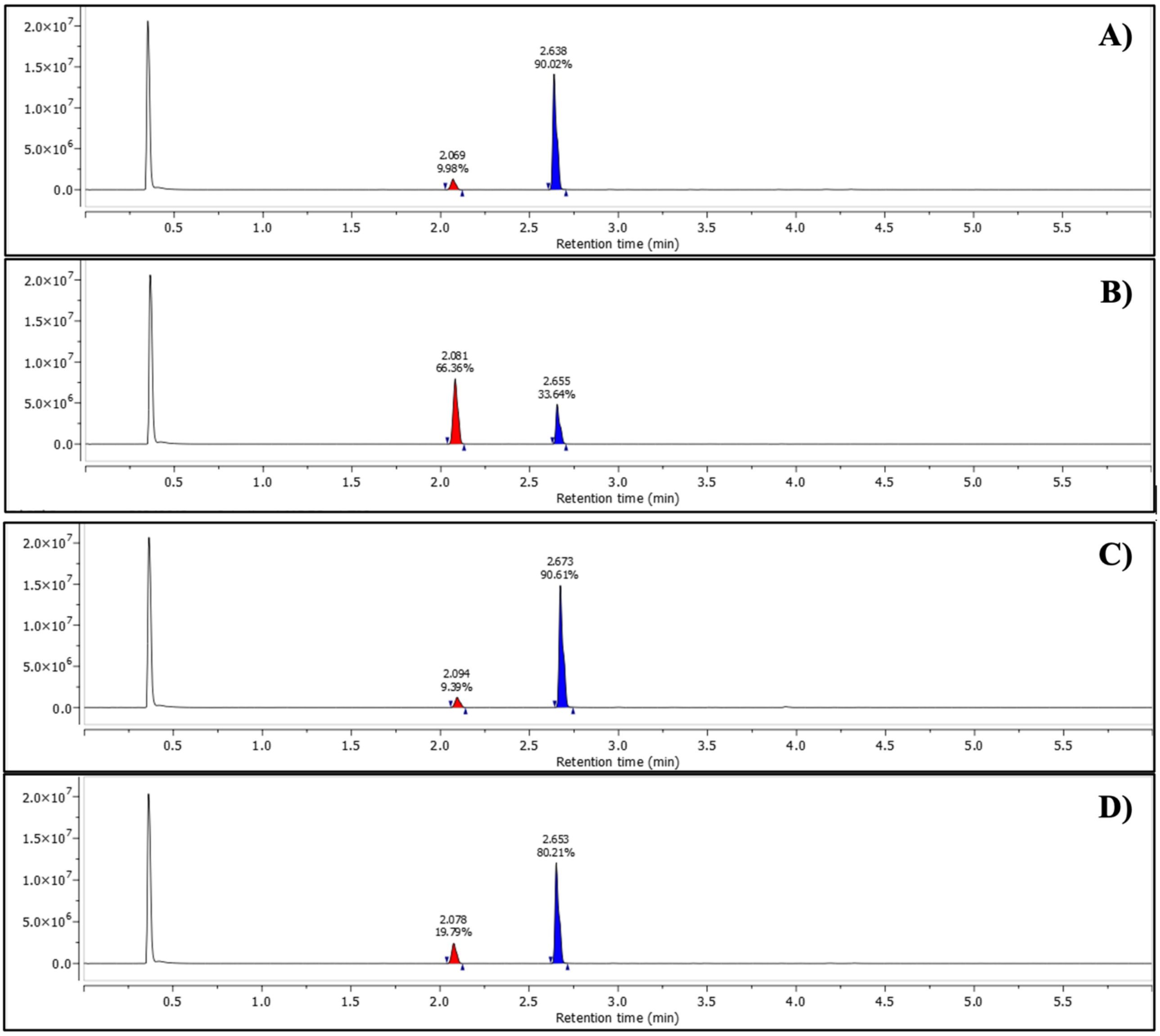

Figure S3. Photostationary states of **photofucose-1** as determined by UPLC-MS at A) benchtop condition, B) after 5 min irradiation at 365 nm, C) after 5 min irradiation at 385-400 nm, and D) after 5 min at 470 nm. The *cis* isomer peak is shown in red, while the *trans* isomer peak is shown in blue.

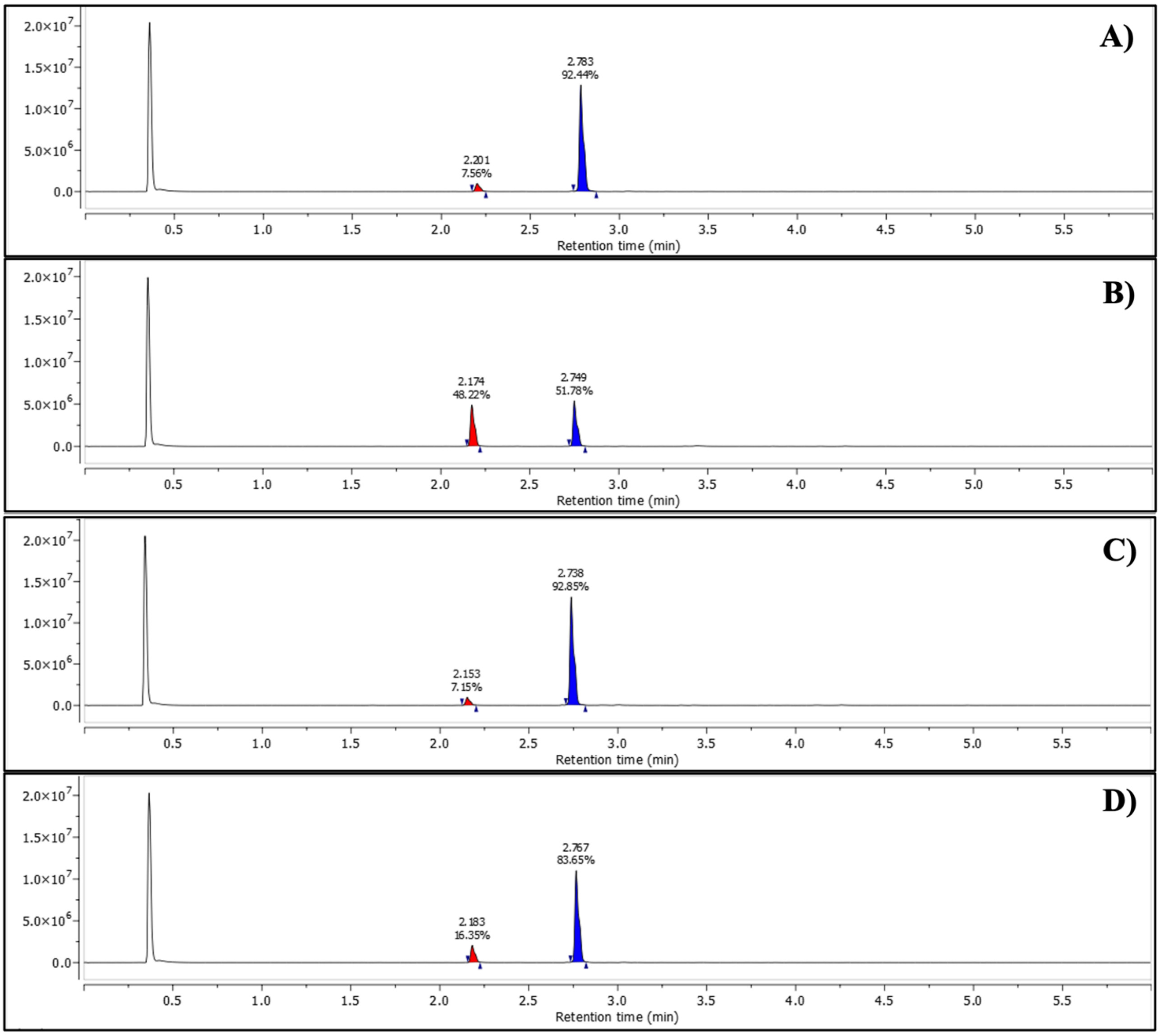

Figure S4. Photostationary states of **photofucose-2** as determined by UPLC-MS at A) benchtop condition, B) after 5 min irradiation at 365 nm, C) after 5 min irradiation at 385-400 nm, and D) after 5 min at 470 nm. The *cis* isomer peak is shown in red, while the *trans* isomer peak is shown in blue.

3. NMR spectra

1. (2*S*,3*S*,4*R*,5*S*,6*R*)-2-methyl-6-(nitromethyl)tetrahydro-2*H*-pyran-3,4,5-triol (Intermediate 1)

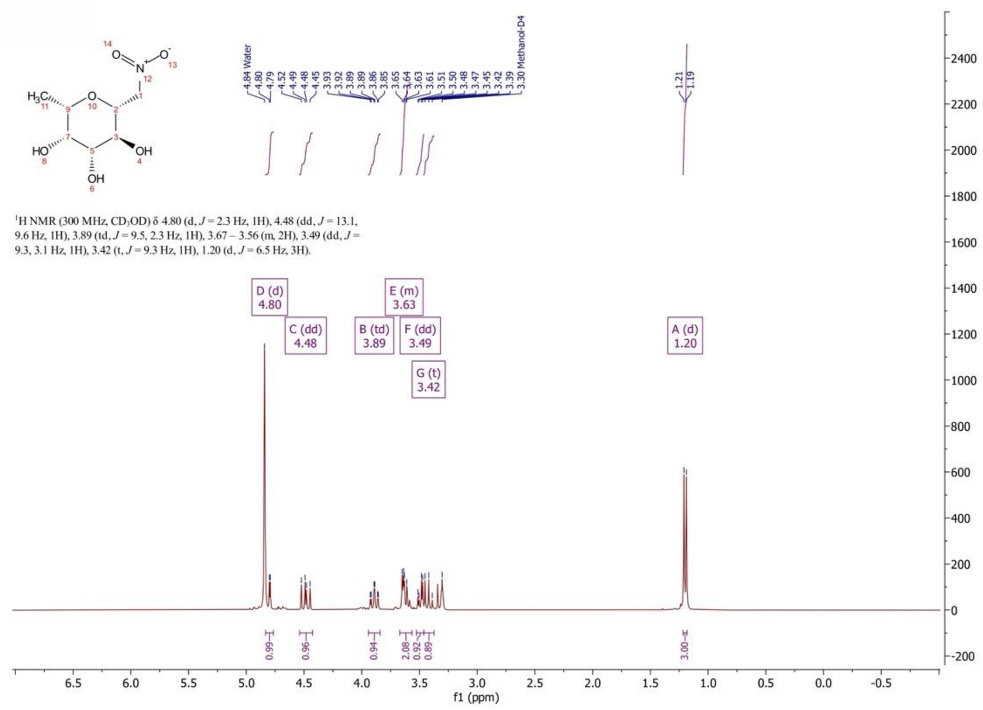

Figure S5. *^{1}H-NMR of Intermediate 1 in CD_3OD.*

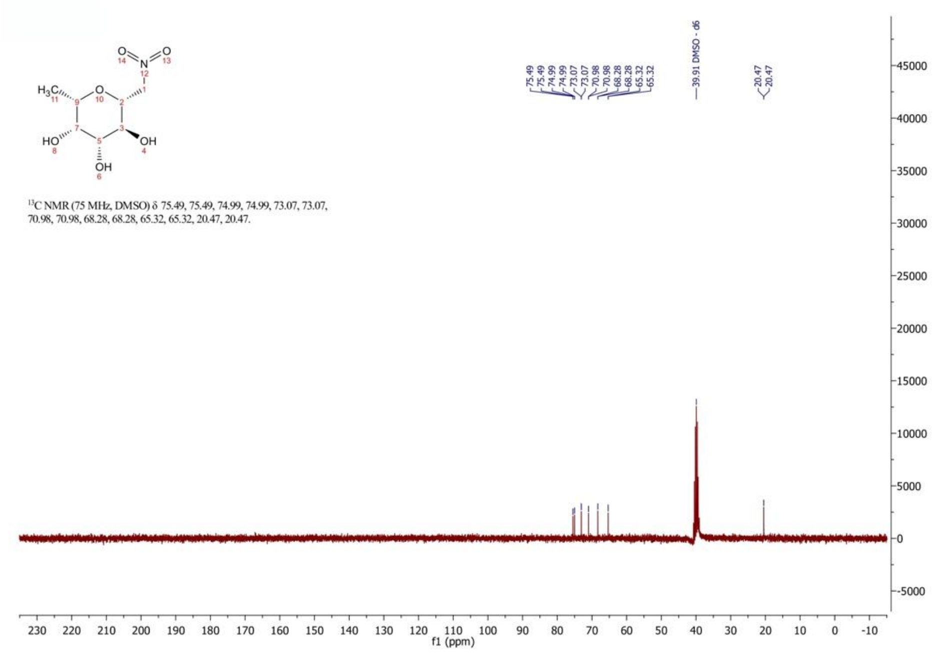

Figure S6. *^{13}C-NMR of Intermediate 1 in DMSO-d_6.*

2. ((2*R*,3*S*,4*R*,5*S*,6*S*)-2-(aminomethyl)-6-methyltetrahydro-2*H*-pyran-3,4,5-triol (Intermediate 2)

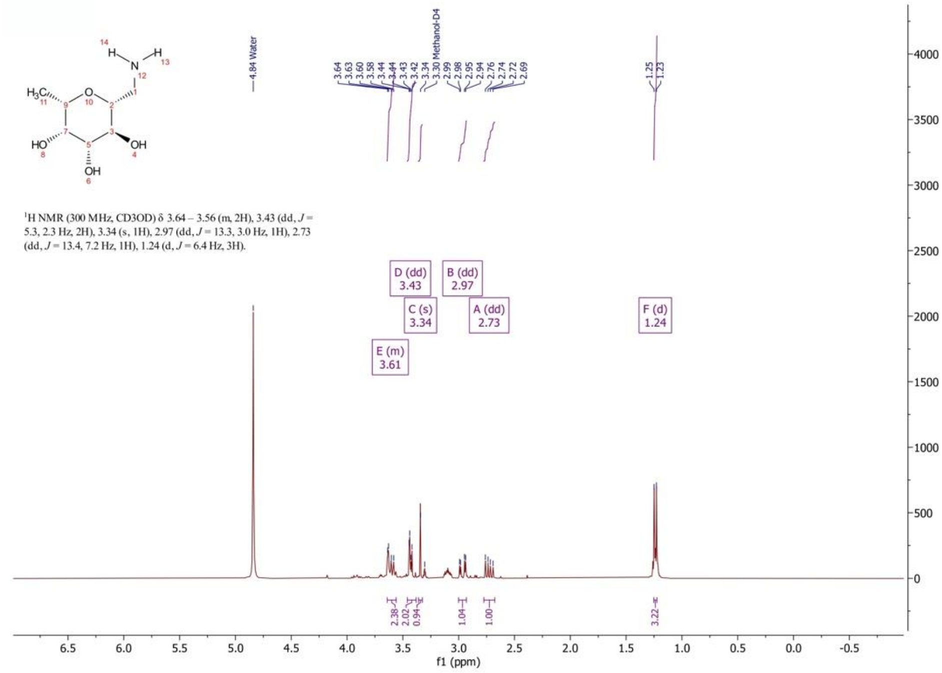

Figure S7. *^{1}H-NMR of Intermediate 2 in CD_3OD.*

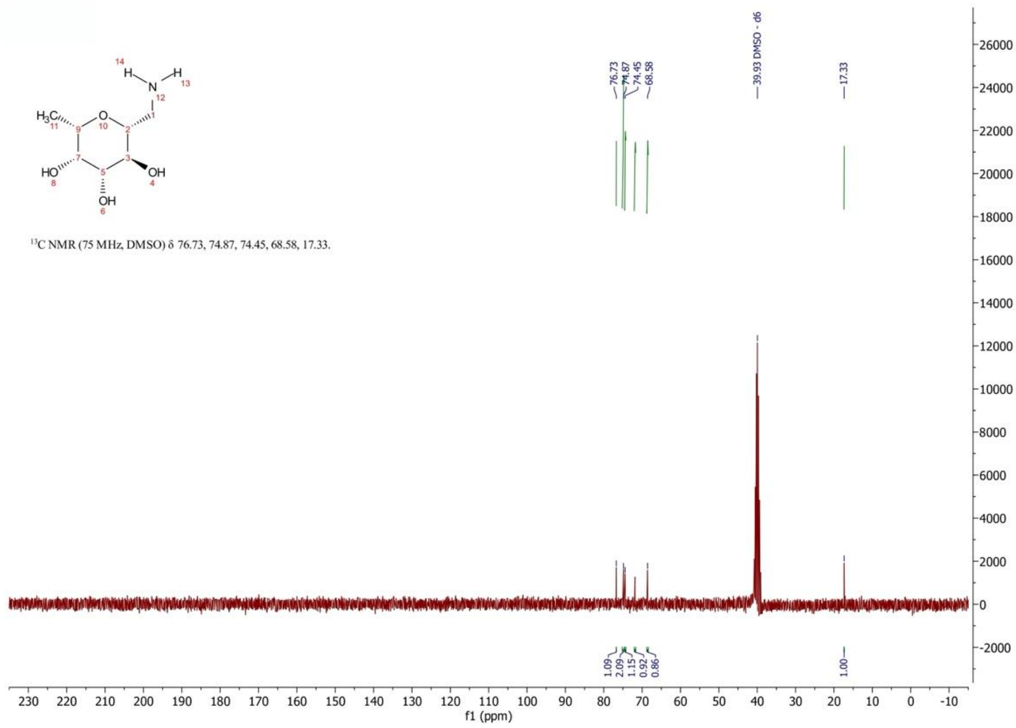

Figure S8. ^{13}C-NMR of *Intermediate 2* in DMSO-d_6

3. (4-((*E*)-phenyldiazenyl)-*N*-(((2*R*,3*S*,4*R*,5*S*,6*S*)-3,4,5-trihydroxy-6-methyltetrahydro-2*H*-pyran-2-yl)methyl)benzamide (**photofucose-1**)

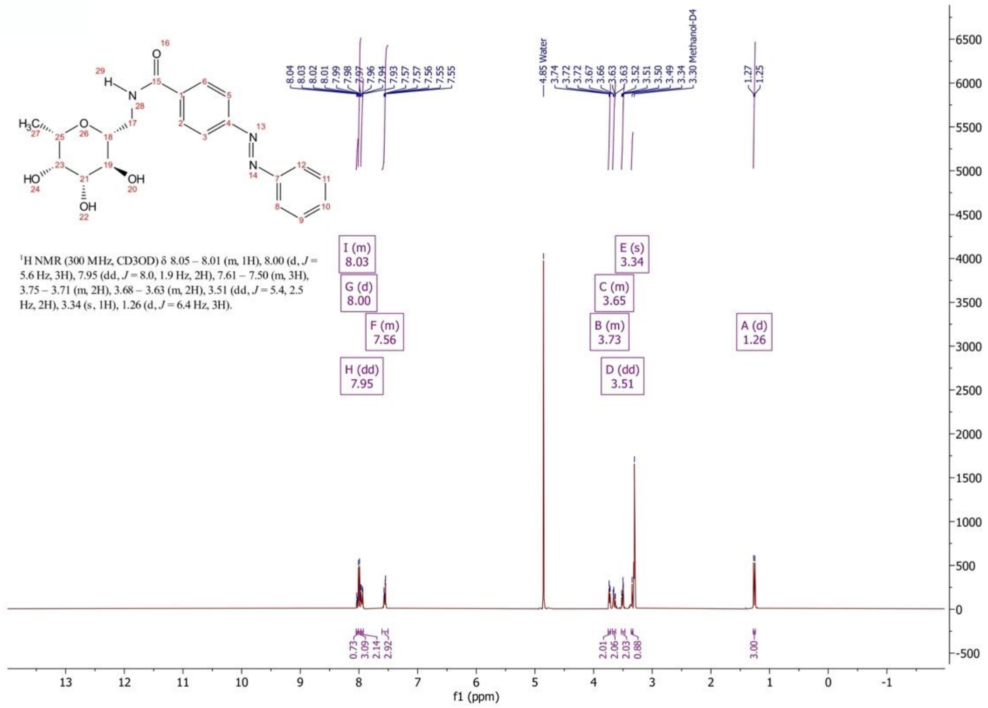

Figure S9. *^{1}H-NMR of **photofucose-1** in CD_3OD.*

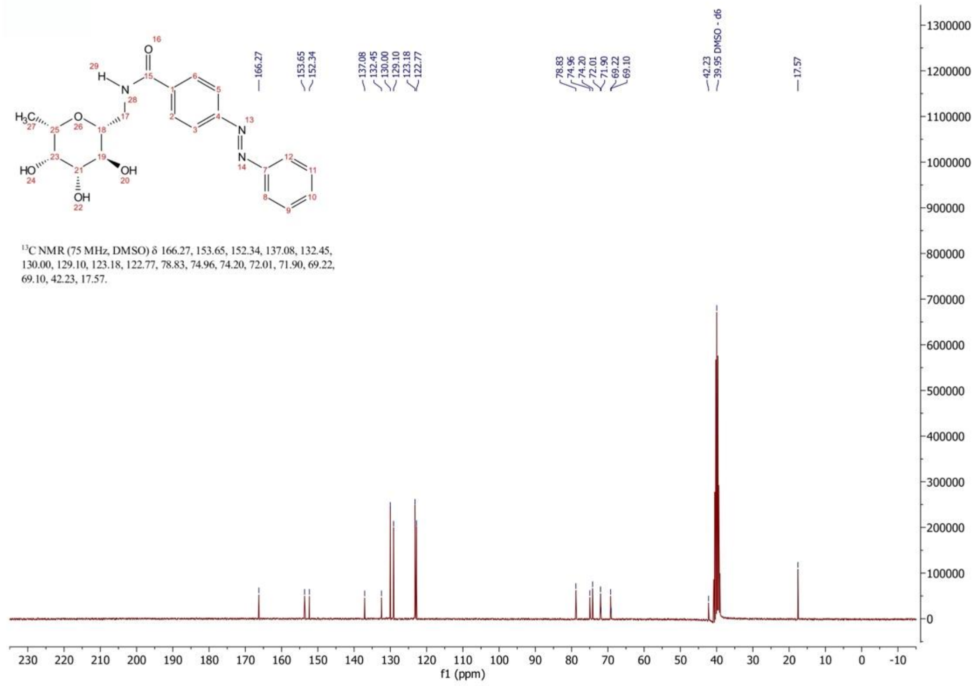

Figure S10. *^{13}C-NMR of **photofucose-1** in DMSO-d_6.*

4. 4-((*E*)-phenyldiazenyl)-*N*-(((2*R*,3*S*,4*R*,5*S*,6*S*)-3,4,5-trihydroxy-6-methyltetrahydro-2*H*-pyran-2-yl)methyl)benzenesulfonamide (**photofucose-2**)

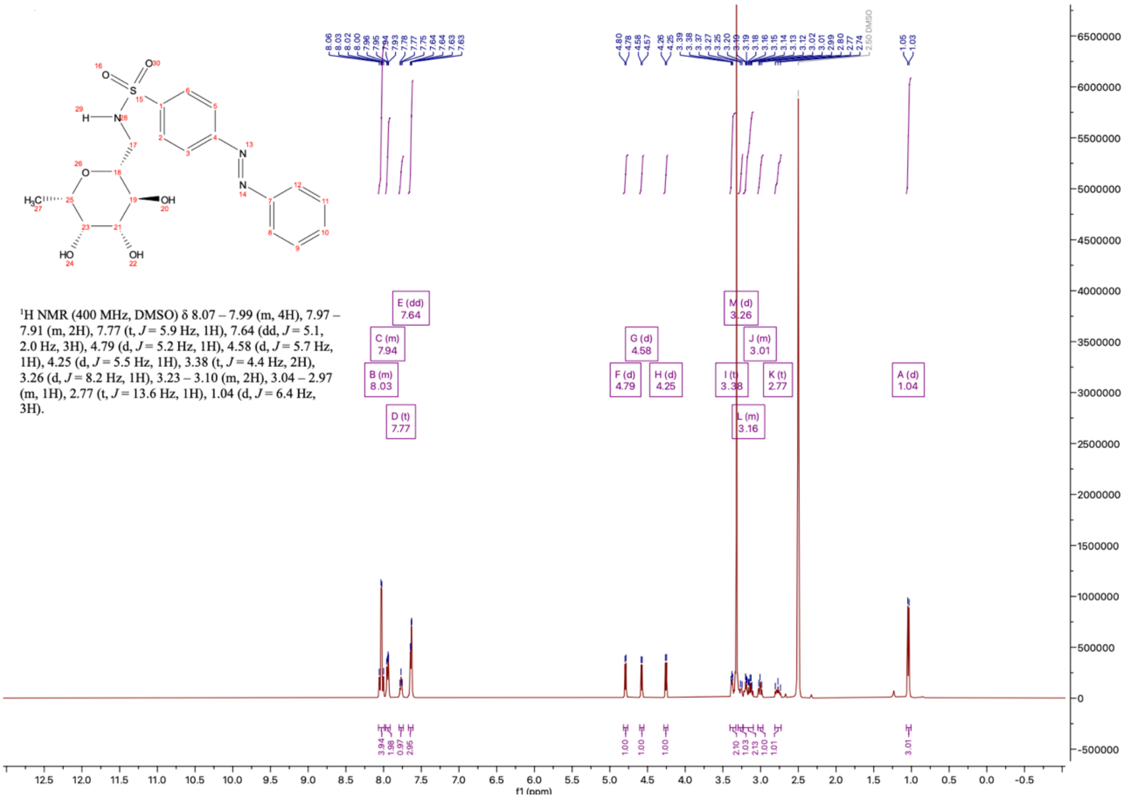

Figure S11. *^{1}H-NMR of* ***photofucose-2*** *in DMSO-d_6.*

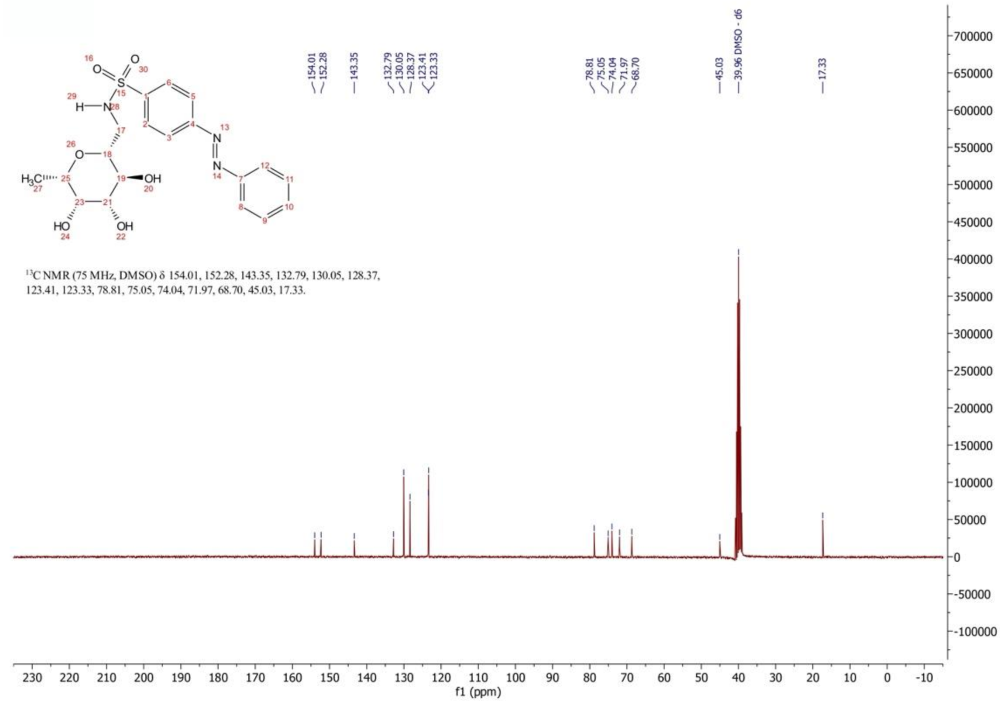

Figure S12. *^{13}C-NMR of* ***photofucose-2*** *in DMSO-d_6.*

4. Mass spectra

High-resolution molecular masses (HRMS) were determined on a Waters Synapt G2-Si hybrid quadrupole time-of-flight (TOF) mass spectrometer equipped with an electron spray ion source (ESI).

1. (4-((*E*)-phenyldiazenyl)-*N*-(((2*R*,3*S*,4*R*,5*S*,6*S*)-3,4,5-trihydroxy-6-methyltetrahydro-2*H*-pyran-2-yl)methyl)benzamide (**photofucose-1**)

Monoisotopic Mass, Even Electron Ions
637 formula(e) evaluated with 2 results within limits (all results (up to 1000) for each mass)
Elements Used:
C: 1-115 H: 1-130 N: 0-10 O: 0-20
1322 Castagna GTA
HRMS_2024_07_144 590 (1.688) Cm (590-565:568)

OSI/FOKL-MS
Synapt G2-Si
1: TOF MS ES+
9.84e+005

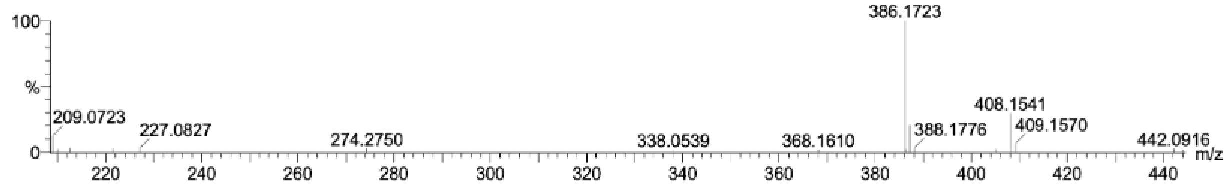

	mDa	PPM	DBE
Minimum:			-0.5
Maximum:	10.0	5.0	200.0

Mass	Calc. Mass	mDa	PPM	DBE	i-FIT	Norm	Conf(%)	Formula
386.1723	386.1729	-0.6	-1.6	15.5	70.0	0.526	59.09	C21 H20 N7 O
	386.1716	0.7	1.8	10.5	70.4	0.894	40.91	C20 H24 N3 O5

Figure S13. *Mass analysis of* ***photofucose-1.***

2. 4-((*E*)-phenyldiazenyl)-*N*-(((2*R*,3*S*,4*R*,5*S*,6*S*)-3,4,5-trihydroxy-6-methyltetrahydro-2*H*-pyran-2-yl)methyl)benzenesulfonamide (**photofucose-2**)

Monoisotopic Mass, Even Electron Ions
652 formula(e) evaluated with 2 results within limits (all results (up to 1000) for each mass)
Elements Used:
C: 1-115 H: 1-130 N: 0-10 O: 0-20 S: 1-1
1323 Castagna GTB
HRMS_2024_07_146 603 (1.729) Cm (603:604-586:589)

OSI/FOKL-MS
Synapt G2-Si
1: TOF MS ES+
1.16e+006

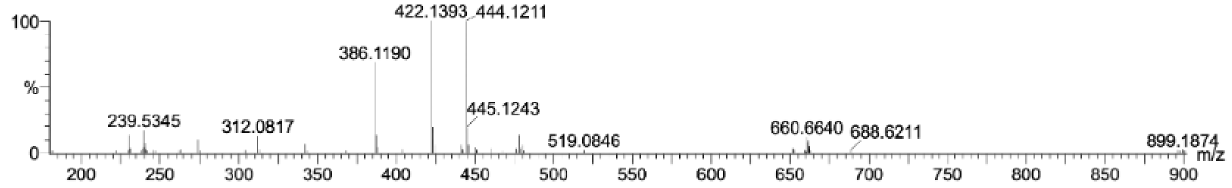

	mDa	PPM	DBE
Minimum:			-0.5
Maximum:	10.0	5.0	200.0

Mass	Calc. Mass	mDa	PPM	DBE	i-FIT	Norm	Conf(%)	Formula
422.1393	422.1399	-0.6	-1.4	14.5	53.7	0.634	53.06	C20 H20 N7 O2 S
	422.1386	0.7	1.7	9.5	53.8	0.756	46.94	C19 H24 N3 O6 S

Figure S14. *Mass analysis of* ***photofucose-2****.*

5. MALDI-TOF

Measurements were recorded with a FLEX-PC autoflex TOF/TOF (Bruker). The acquisition operation mode was Linear, polarity voltage POS. The number of shots was 500. Protein and formic acid (one sample with 1 µL and one with 0.5 µL) were mixed. One drop of this solution was mixed with matrix SA (sinapinic acid) and let it solidify. The concentration of the protein was 10 mg/mL.

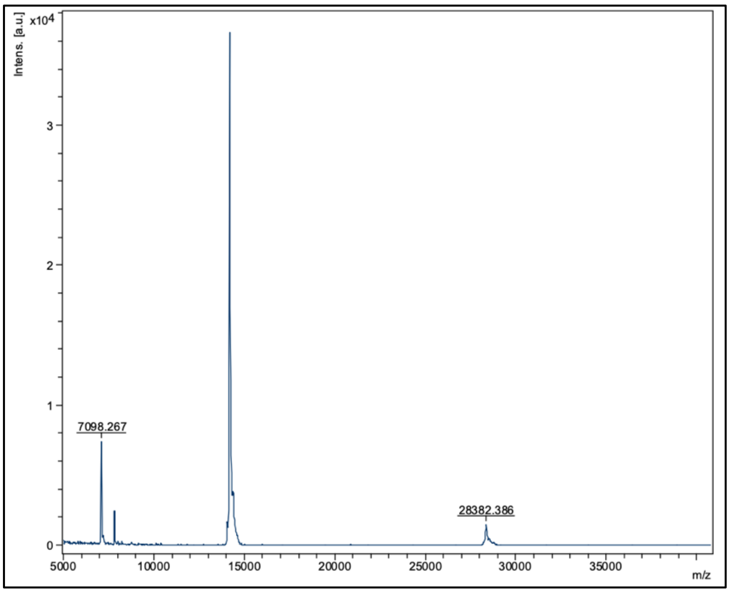

Figure S15. MALDI-TOF analysis of LecB-His. This value was obtained with a 10 mg/mL protein concentration, formic acid, and sinapinic acid (SA) as the matrix.

6. Illumination protocols

To achieves photoconversion 1,5 mL of the solution in cuvette was irradiated in a closed reflective mirror box for 3-5 minutes, using the led arrays at the chosen wavelength. To determine the qualitative conversion of the compound, the cuvette was then put in the UV-vis spectrometers. Measurements were performed at 100 mm from the LED along the emission axis.

Wavelength (nm)	Colour	Intensity	Instrument for the analysis
365	Blue-purple	0.7 mW/cm^2	NanoDrop One^C
385-400	Purple	3.0 mW/cm^2	NanoDrop One^C
470	Blue	4.0 mW/cm^2	NanoDrop One^C
525	Green	1.9 mW/cm^2	NanoDrop One^C
630	Red	2.4 mW/cm^2	NanoDrop One^C

TableS1.*Intensity of the lights source used for the analysis.*

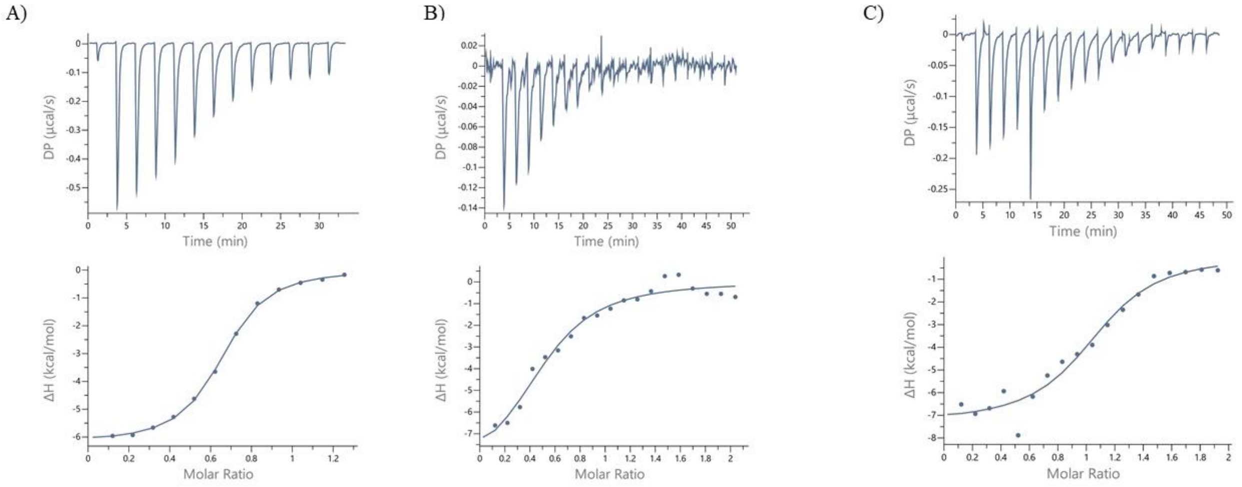

Ligand	K_d [µM]	N (sites)	−ΔG [kcal/mol]	−ΔH [kcal/mol]	TΔS [kJ/mol]
l-fucose	1.48 ± 0.116	0.630 ± 0.004	-7.95	-6.12 ± 0.083	-1.83

Ligand	K_d [µM]	N (sites)	−ΔG [kcal/mol]	−ΔH [kcal/mol]	TΔS [kJ/mol]
l-fucose	1.61 ± 0.133	0.629 ± 0.004	-7.9	-6.23 ± 0.091	-1.67

Ligand	K_d [µM]	N (sites)	−ΔG [kcal/mol]	−ΔH [kcal/mol]	TΔS [kJ/mol]
l-fucose	2.04 ± 0.109	1.05 ± 0.063	-7.76	-7.34 ± 0.88	-0.428

Figure S16. ITC measurements of *L-fucose* (n=3)

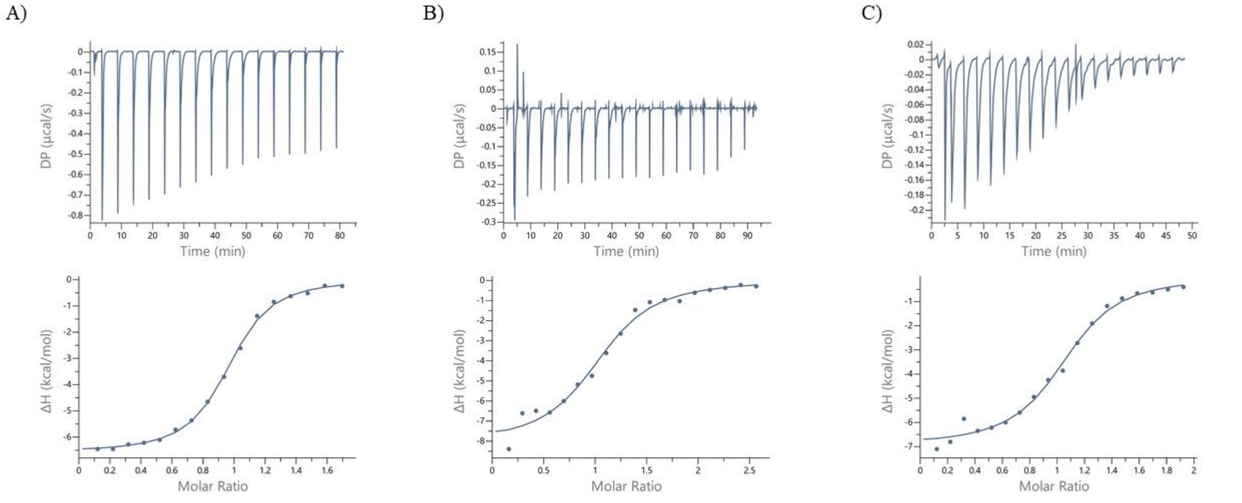

Ligand	K_d [µM]	N (sites)	−ΔG [kcal/mol]	−ΔH [kcal/mol]	TΔS [kJ/mol]
trans-photofucose-2	1.61 ± 0.158	0.937 ± 0.007	-7.91	-6.61 ± 0.109	-1.29

Ligand	K_d [µM]	N (sites)	−ΔG [kcal/mol]	−ΔH [kcal/mol]	TΔS [kJ/mol]
trans-photofucose-2	2.29 ± 0.839	1.04 ± 0.013	-7.7	-8.12 ± 0.656	-0.418

Ligand	K_d [µM]	N (sites)	−ΔG [kcal/mol]	−ΔH [kcal/mol]	TΔS [kJ/mol]
trans-photofucose-2	1.56 ± 0.407	1.04 ± 0.027	-7.92	-6.98 ± 0.369	-0.944

Figure S17. ITC measurements of *trans-**photofucose-2** (n=3)*

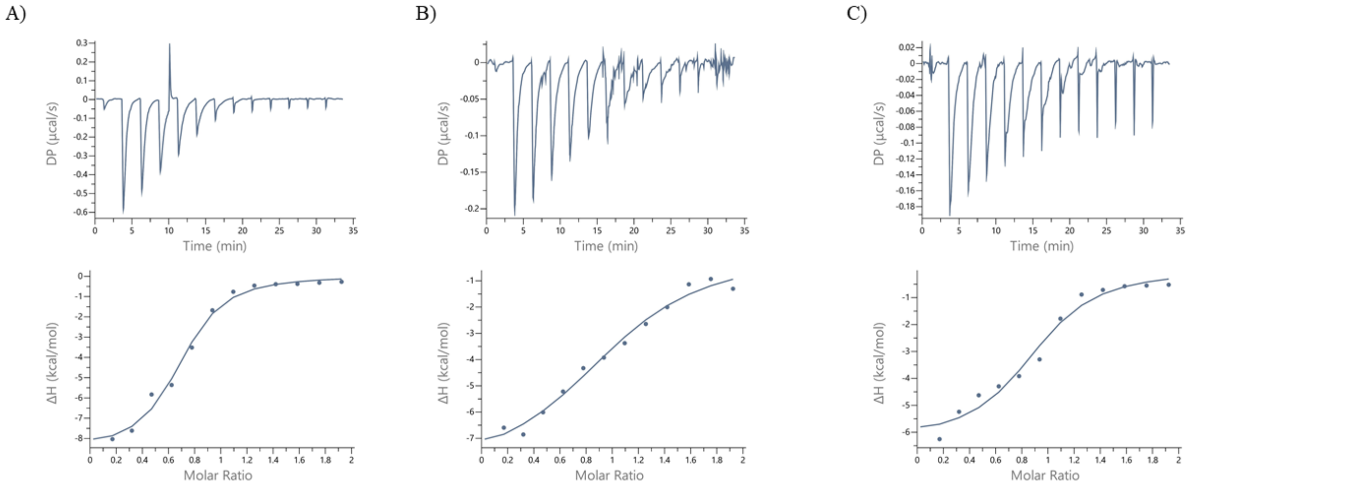

Ligand	K_d [µM]	N (sites)	−ΔG [kcal/mol]	−ΔH [kcal/mol]	TΔS [kJ/mol]
cis-photofucose-2	3.54 ± 1.33	0.660 ± 0.027	-7.44	-8.62 ± 0.654	-1.18

Ligand	K_d [µM]	N (sites)	−ΔG [kcal/mol]	−ΔH [kcal/mol]	TΔS [kJ/mol]
cis-photofucose-2	7.7 ± 4.45	1.01 ± 0.116	-7.02	-8.32 ± 1.8	-1.3

Ligand	K_d [µM]	N (sites)	−ΔG [kcal/mol]	−ΔH [kcal/mol]	TΔS [kJ/mol]
cis-photofucose-2	2.9 ± 1.8	0.883 ± 0.06	-7.56	-6.31 ± 0.963	-1.24

Figure S18. ITC measurements of *cis-**photofucose-2*** *(n=3)*

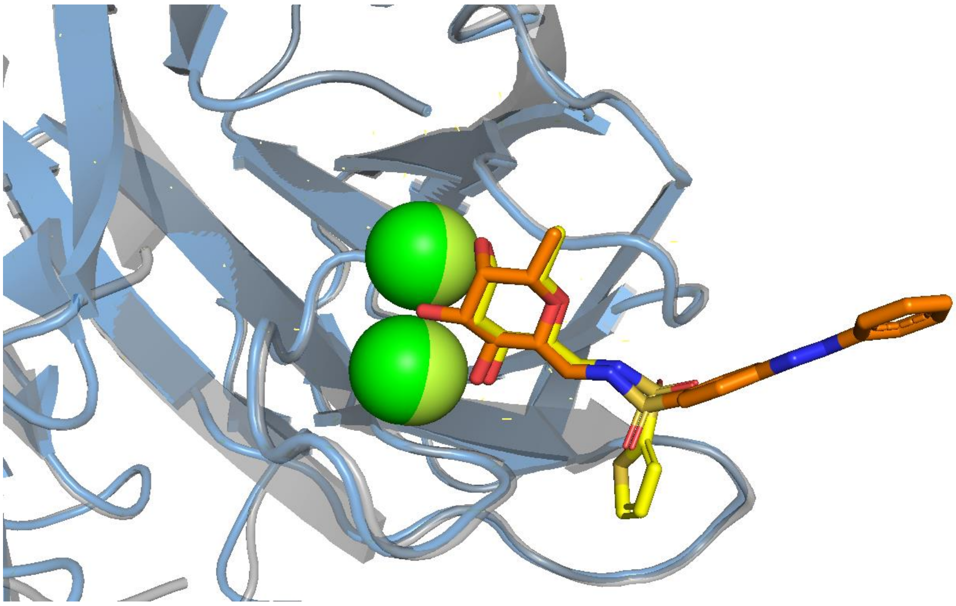

Figure S19. Structural superposition of fucose binding site of LecB PDB:9HD4 vs PDB:5MAY (Gray:5MAY backbone; light blue: 9HD4 backbone, green spheres: calcium ions of 9HD4, light green spheres: calcium ions of 5MAY, red: oxygens, blue: nitrogen, orange: **photofucose-2**, yellow: 2-Thiophenesulfonamide-N-(beta-L-fucopyranosyl methyl). The overall structural alignment shows no significant differences for the orientation and the binding mode of the fucose moiety within the binding pocket.
